# Conserved paradoxical relationships among the evolutionary, structural and expressional features of KRAB zinc-finger proteins reveal their special functional characteristics

**DOI:** 10.1186/s12860-021-00346-w

**Published:** 2021-01-22

**Authors:** Pan Shen, Aishi Xu, Yushan Hou, Huqiang Wang, Chao Gao, Fuchu He, Dong Yang

**Affiliations:** 1 State Key Laboratory of Proteomics, Beijing Proteome Research Center, National Center for Protein Sciences (Beijing), Beijing Institute of Lifeomics, Beijing, 102206 China; 2 Animal Sciences College of Jilin University, Changchun, 130062 China

**Keywords:** KZFP, Evolutionary age, Structure, Expression, Function

## Abstract

**Background:**

One striking feature of the large KRAB domain-containing zinc finger protein (KZFP) family is its rapid evolution, leading to hundreds of member genes with various origination time in a certain mammalian genome. However, a comprehensive genome-wide and across-taxa analysis of the structural and expressional features of KZFPs with different origination time is lacking. This type of analysis will provide valuable clues about the functional characteristics of this special family.

**Results:**

In this study, we found several conserved paradoxical phenomena about this issue. 1) Ordinary young domains/proteins tend to be disordered, but most of KRAB domains are completely structured in 64 representative species across the superclass of Sarcopterygii and most of KZFPs are also highly structured, indicating their rigid and unique structural and functional characteristics; as exceptions, old-zinc-finger-containing KZFPs have relatively disordered KRAB domains and linker regions, contributing to diverse interacting partners and functions. 2) In general, young or highly structured proteins tend to be spatiotemporal specific and have low abundance. However, by integrated analysis of 29 RNA-seq datasets, including 725 samples across early embryonic development, embryonic stem cell differentiation, embryonic and adult organs, tissues in 7 mammals, we found that KZFPs tend to express ubiquitously with medium abundance regardless of evolutionary age and structural disorder degree, indicating the wide functional requirements of KZFPs in various states. 3) Clustering and correlation analysis reveal that there are differential expression patterns across different spatiotemporal states, suggesting the specific-high-expression KZFPs may play important roles in the corresponding states. In particular, part of young-zinc-finger-containing KZFPs are highly expressed in early embryonic development and ESCs differentiation into endoderm or mesoderm. Co-expression analysis revealed that young-zinc-finger-containing KZFPs are significantly enriched in five co-expression modules. Among them, one module, including 13 young-zinc-finger-containing KZFPs, showed an ‘early-high and late-low’ expression pattern. Further functional analysis revealed that they may function in early embryonic development and ESC differentiation via participating in cell cycle related processes.

**Conclusions:**

This study shows the conserved and special structural, expressional features of KZFPs, providing new clues about their functional characteristics and potential causes of their rapid evolution.

**Supplementary Information:**

The online version contains supplementary material available at 10.1186/s12860-021-00346-w.

## Background

KRAB domain-containing zinc finger protein (KZFP) family is the largest family of transcription factors in mammals [1]. For example, there are 387 KZFP-coding genes in the human genome. Generally, KZFP contains a KRAB domain and a C-terminal C2H2 zinc finger array with DNA-binding potential (Fig. S1 in Additional file 1). The specificity of the binding sequence is depended mainly on three key amino acids within each C2H2 zinc finger (at positions 6, 3 and − 1 of the C2H2 helix), and some contacts being established with the secondary strand via the amino acid at position 2 [2, 3]. Both C2H2 zinc finger and KRI (KRAB Interior) motif, which is the ancestor of KRAB domain, are old motifs, appearing widely across animals, plants and fungi [4]. However, these two kinds of motifs did not appear in the same protein during the lengthy process of evolution until their ‘marriage’ in the last common ancestor of coelacanths and tetrapods [5] about 400 million years ago. After that, the KZFP family expanded and diverged quickly, especially during the evolution of mammals [1, 2, 6].

As the result of the rapid evolution of this family, KZFP genes with various evolutionary ages exist in the current genomes [2, 5, 7]. The evolutionary age of KZFP genes can be represented by the last common ancestor of the species containing the homologous KZFPs determined by the similarity of the full KZFP protein sequence [2, 6]. In addition, due to the rapid divergence of the key amino acids in C2H2 zinc fingers [2, 5, 8, 9], which determine their DNA binding specificity, the evolutionary age can also be measured by the divergence time of the key amino acids in C2H2 zinc fingers [5]. Thus, these two types of evolutionary age of KZFPs are included in this study. KZFPs in a certain species can be divided into several classes according to their evolutionary age grades. Exploring the functional characteristics of KZFPs with different evolutionary ages is of great significance to fully understand the mechanism of rapid evolution of this large family.

Structural and expressional features are closely related to the functional characteristics of proteins. The protein intrinsic disorder degree, one of the key structural features, affects protein function and protein-protein interaction network [10, 11], and the variation of protein structural disorder may cause many diseases [12, 13]. On the other hand, the spatiotemporal expression pattern may provide important clues of protein functions. Thus, it’s essential to explore the structural disorder degrees and expressional features of KZFPs with different origination time to deep understand the functional requirements of this large divergent family during evolution. However, the answer to this issue is unclear so far. At present, there is hardly any systematic understanding on the structural characteristics (especially protein/domain disorder) of KZFP family. Part of the expression patterns of KZFPs in a series of biological samples, including embryonic stem cells (ESCs) [14, 15], developmental brains [16], adult organs, tissues or cells [5, 16–19], have been analyzed in previous studies. However, these studies focused on the expression patterns of KZFPs only in a few samples and species, and most of them didn’t closely link the expression patterns with the evolutionary and structural characteristics of KZFPs.

In this study, we systematically explored the relationships between evolutionary age and structural disorder features of KZFPs, the expression width or expression level across early embryonic development, ESCs differentiation, embryonic and adult organs, tissues in 7 mammals. In total, 29 RNA-seq datasets, including 725 samples were involved in the analysis. Some conserved paradoxical phenomena were observed in these analyses, providing new clues about their functional characteristics and the potential causes of the rapid evolution of this large family.

## Results

### KRAB domains are evolutionarily young, but most of them are completely structured

**Fig. 1:**
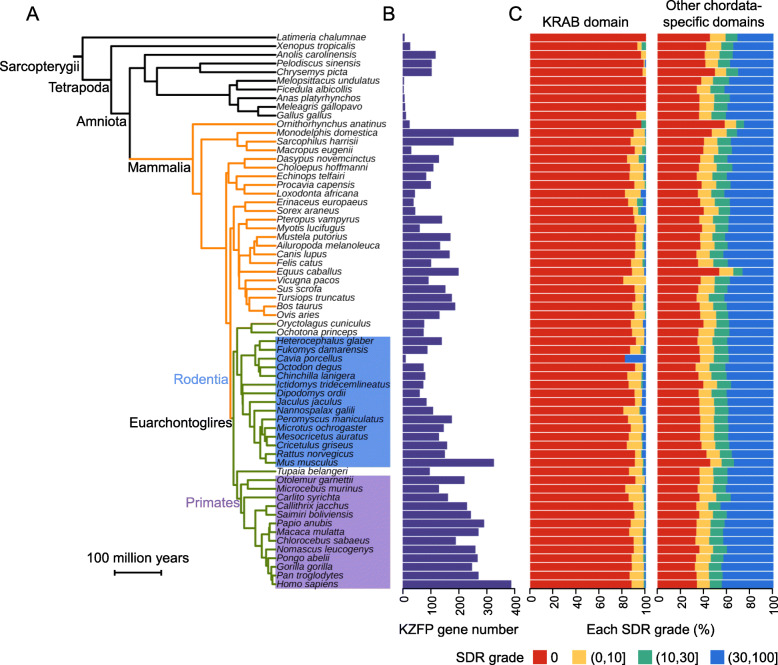
The structural disorder degree of KRAB domains in the 64 representative species. **a** The species tree shows the evolutionary relationship among 64 species, including one species of coelacanth and 63 species of tetrapod. Among tetrapods, 54 mammals, including 15 rodents and 13 primates, were shown. **b** KZFP gene number in 64 species. **c** Percentages of each SDR grade of KRAB domains and other chordata-specific domains in each of the 64 species

For the protein domains in chordates, those originating after the origination of the common ancestor of chordates are usually regarded as evolutionarily young domains [20, 21]. In general, young domains tend to be highly disordered (Fig. 1 & Additional file 2). KRAB domains, originating in the last common ancestor of coelacanths and tetrapods, are definitely young domains. Thus, KRAB domains should be highly disordered according to the existing knowledge. To test this hypothesis, we compared the structural disordered ratio (SDR) of KRAB domain with other chordates-specific domains in 64 species. To our surprise, on average, 89.2% of KRAB domains in 64 species are completely structured, that is, the SDR values of them are zero (Fig. 1 & Additional file 2). However, only 37.6% (mean) of other chordata-specific domains are completely structured, whereas 38.6% (mean) of them are highly disordered domains (Fig. 1 & Additional file 2). These results indicate that there is an evolutionarily conserved pattern that most KRAB domains are completely structured.

### Most KZFPs tend to be highly structured with exception that old-zinc-finger-containing KZFPs tend to be relatively disordered

**Fig. 2:**
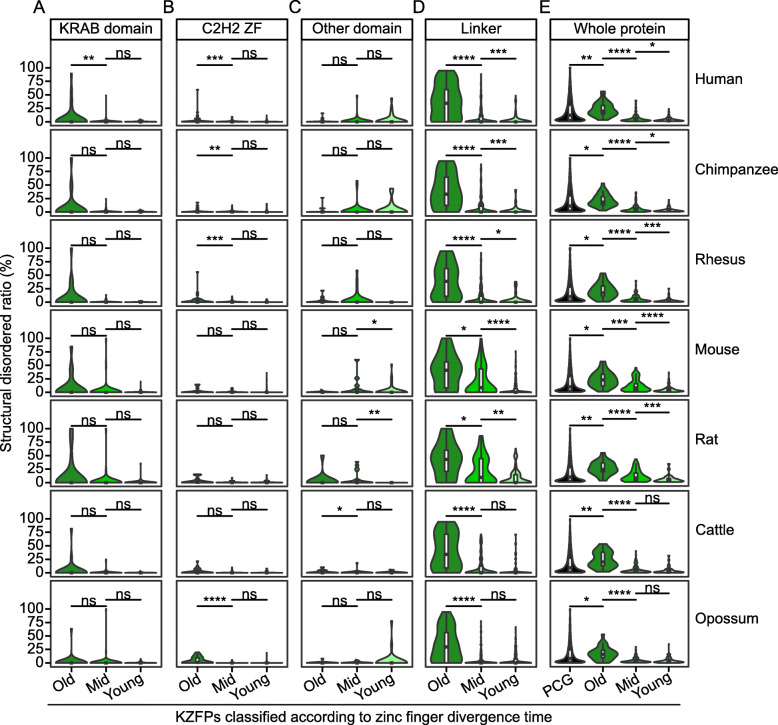
The structural disorder degree of different regions of KZFPs with different zinc finger divergence time grades in 7 mammals. **a-d** Box-plots of SDR values of KRAB domain (**a**), C2H2 zinc fingers (**b**), other domains (**c**) and linker region (**d**) in KZFPs with different zinc finger divergence time grades in 7 mammals. **e** Comparison of SDR values of KZFPs with different zinc finger divergence time grades and other proteins in 7 mammals. For box-plots, the values of upper and lower quartiles are indicated as upper and lower edges of the box, and the median values of median are indicated as a bar in the box. The number of KZFPs in each category is in Additional file 10. The differences of SDR value between different categories are examined by Mann–Whitney U test. The corrected *P* values are shown in the top of each panel. ns: *p* > 0.05, *: *p* < 0.05, **: *p* < 0.01, ***: *p* < 0.001, ****: *p* < 0.0001

Although the vast majority of KRAB domains are completely structured, there are still some KRAB domains containing disordered amino acid residues. Since the core purpose in this study is to explore the difference of characteristics among the KZFPs with various origination time, we investigated whether there are differences in the SDR of KRAB domains in KZFPs with different gene age. By comparing the SDR values of KRAB domains in KZFPs with different gene age grades in 7 mammals, including human (*Homo sapiens*), chimpanzee (*Pan troglodytes*), rhesus (*Macaca mulatta*), mouse (*Mus musculus*), rat (*Rattus norvegicus*), cattle (*Bos taurus*) and opossum (*Monodelphis domestica*), we found that there are no significant differences between KZFPs with different gene age grades (Figure S2A in Additional file 1, Additional file 3). Considering that the regulatory specificities of KZFPs are mainly determined by the zinc fingers binding to the target sequences, we also analyzed the SDR values of KRAB domains in the KZFPs with different zinc finger divergence times. Interestingly, we found that the KRAB domains in old-zinc-finger-containing KZFPs tend to be disordered (Fig. 2a).

To further analyze the structural characteristics of KZFPs, we calculated the SDR values of multiple regions, including C2H2 zinc fingers, other domains and the linker regions (the non-domain region between KRAB domain and C2H2 zinc fingers). Similarly, we found that there were no significant differences in disorder degree of those regions in KZFPs among different gene age grades (Figure S2B-S2D in Additional file 1, Additional file 3). The SDR values of them are all relatively small. Consequently, the whole protein of KZFPs with each gene age grade all tend to be highly structured (Figure S2E in Additional file 1, Additional file 3); whereas the proteins encoded by young protein-coding genes (PCGs) have a higher disordered degree in all 7 mammals (Figure S2F in Additional file 1, Additional file 4). In terms of zinc finger divergence time grade, we also found that the C2H2 zinc fingers and other domains tend to be highly structured in all grades (Fig. 2b & c). Interestingly, the linker regions in old-zinc-finger-containing KZFPs are significantly more disordered in all 7 mammals (Fig. 2d), making KZFPs more flexible. These disordered regions in old-zinc-finger-containing KZFPs (about 10% in 7 mammals) lead to the higher disorder degree of the whole proteins, compared with other proteins; whereas most of KZFPs encoding relatively younger zinc fingers (about 90% in 7 mammals) tend to be highly structured (Fig. 2e, Additional file 3).

### KZFP genes tend to be expressed ubiquitously with a medium level regardless of evolutionary age and structural disorder degree

Generally speaking, young genes tend to be expressed spatiotemporal specifically [20–23]. Thus, we supposed that KZFP genes should also tend to be specifically expressed because each KZFP has a young KRAB domain (Chordata-specific), and according to the full protein, about half of them are mammalian-specific genes in 7 mammals.

To validate this hypothesis, the gene expression data from 29 RNA-seq datasets, including 725 samples from early development stage to adult across 7 mammals, were collected (see Methods) and the expression width of each gene was calculated. The samples included the embryos of early development, different time points during ESCs differentiation into three germ layers and the subsequent terminal-differentiated cells, embryonic development of various organs and various adult tissues or organs. The number of samples in which a certain gene expressed was defined as the expression width of the gene.

**Fig. 3:**
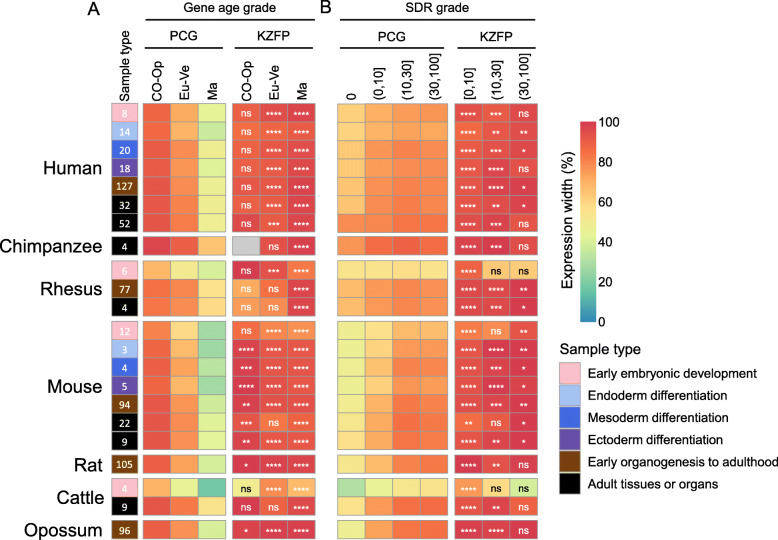
The expression width of KZFP genes with different gene age grades or SDR grades in mammals. Samples in 7 mammals, including early embryonic development, three directions of ESCs differentiation, organs from early organogenesis to adulthood and adult tissues or organs were used in the analysis. Heatmap shows the percentage of expression width of each gene age grade (**a**) or SDR grade (**b**) in each sample. The number in the box on the left represents the total number of samples. The number of KZFPs and PCGs in each category is in Additional file 10. The category with gene number 0 is displayed in gray. The differences of expression width between KZFP genes and PCGs with different categories are examined by Mann–Whitney U test. The corrected P values are shown in the top of each panel. ns: *p* > 0.05, *: *p* < 0.05, **: *p* < 0.01, ***: *p* < 0.001, ****: *p* < 0.0001. Gene age grade: CO − Op, Cellular organisms−Opisthokonta; Eu-Ve, Eumetazoa−Vertebrata; Ma, Mammalia

It is obvious that young genes (Mammalian-specific) tend to be expressed at specific timepoints or spaces, and old genes tend to be wildly expressed (Fig. 3a). To our surprise, although about half KZFP genes are young genes, both old and young KZFPs are widely expressed, compared with other PCGs (Fig. 3a, Additional file 5). In addition, we also analyzed the expression patterns of KZFP genes with different zinc finger ages and similar results were obtained (Figure S3A in Additional file 1, Additional file 5). Comparing the young-zinc-finger-containing KZFPs with others, we found that the expression width of young-zinc-finger-containing KZFPs are relatively narrower (Figure S3A in Additional file 1, Additional file 5).

The variable and diverse conformations of intrinsic disordered proteins make them have the potential to interact more proteins [24], while highly structured proteins should only interact to a specific protein because of the monotonous and unchangeable regulatory mode based on their changeless conformation [10]. Thus, we hypothesized that the expression width of disordered proteins is greater than that of structured proteins (*e. g.*, KZFPs). For PCGs, genes encoding completely structured proteins tend to be expressed spatiotemporal specifically compared with disordered proteins (Fig. 3b). However, almost all KZFPs tend to be widely expressed regardless of disorder degree. These results show that KZFP genes tend to be ubiquitously expressed regardless of gene age, zinc finger divergence time and SDR, suggesting there are wide functional requirements of KZFPs in various states.

**Fig. 4:**
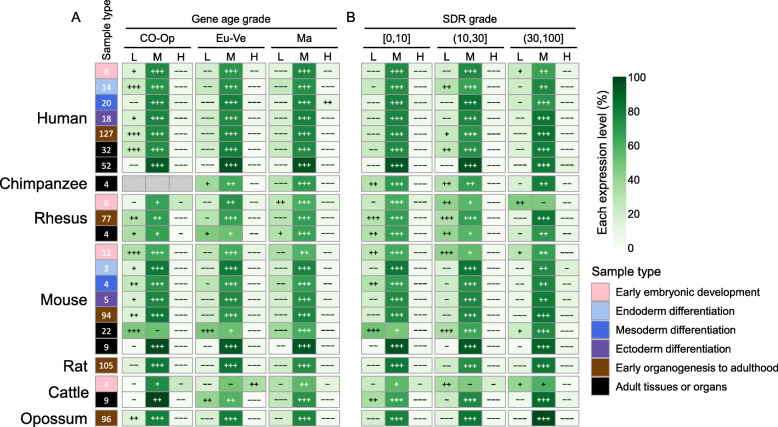
The expression level of KZFP genes with different gene age grades or SDR grades in mammals. Samples in 7 mammals, including early embryonic development, three directions of ESC differentiation, organs from early organogenesis to adulthood and adult tissues or organs were used in the analysis. Heatmap shows the proportion of three expression level grades of KZFPs with each gene age (**a**) or SDR grade (**b**) grade. The number in the box on the left represents the total number of samples. The number of KZFPs and PCGs in each category is in Additional file 10. The category with gene number 0 is displayed in gray. The over/under representation strength shows KZFP genes relative to PCGs with different expression levels in each gene age grade or SDR grade in each sample. Over representation strength: +, *P* ≥ 0.05; ++, *P* < 0.05; +++, *P* < 10^− 10^. Under representation strength: −, *P* ≥ 0.05; −−, *P* < 0.05; −−−, *P* < 10^− 10^. Gene age grade: CO − Op, Cellular organisms−Opisthokonta; Eu-Ve, Eumetazoa−Vertebrata; Ma, Mammalia. Expression level: L, low-abundant level; M, medium-abundant level; H, high-abundant level

Previous studies have shown that old genes often have higher expression level than young genes [22, 23, 25]. To verify whether this trend is also valid in KZFPs, we next analyzed the expression pattern of KZFP genes from the quantitative perspective. First of all, we used the upper and lower quartiles of expression abundances of all expressed genes to divide them into three expression levels (L, low-abundant level; M, medium-abundant level; H, high-abundant level) in each dataset (see Methods). The over/under -representation analysis of KZFP genes relative to PCGs in each gene age grade or SDR grade revealed that KZFP genes are over-represented in the medium-level class in almost all age grades (Fig. 4a) or disorder degrees (Fig. 4b) across 7 mammals, indicating the results of wide expression of KZFP genes are credible, instead of low-level noisy signals. Additionally, we counted the proportion of three expression level grades of KZFP genes with each zinc finger divergence time grade, and found that most KZFP genes are also in medium abundance in each zinc finger divergence time grade (Figure S3B in Additional file 1). These results show that KZFPs tend to be ubiquitously expressed with medium abundance regardless of gene age, zinc finger divergence time and SDR degree across 7 mammals, indicating that there is a conserved expression pattern of KZFP genes in mammals.

### The specific expression pattern of KZFPs

**Fig. 5:**
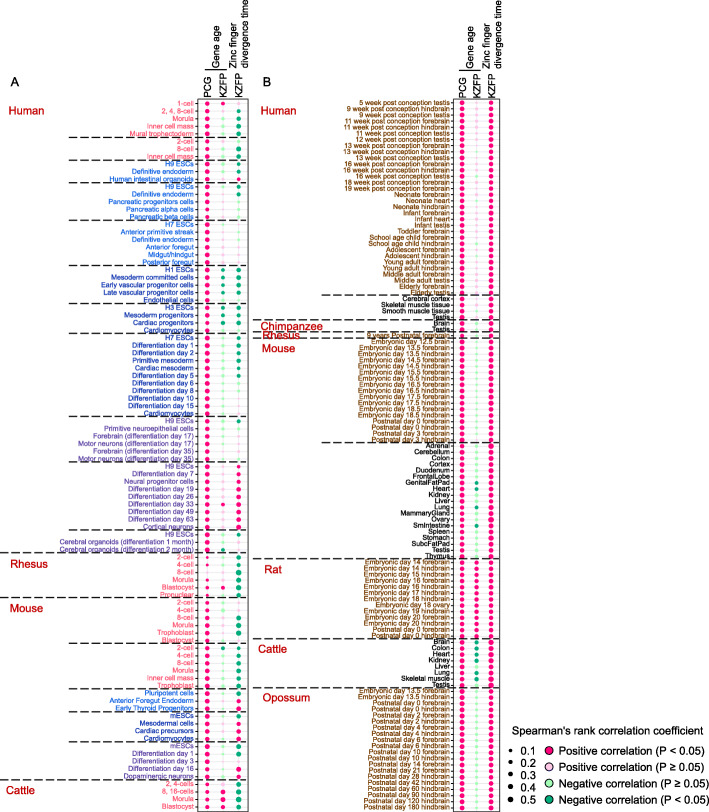
The correlation between the evolutionary age and expression abundance of genes in mammals. **a** Heatmap shows spearman’s rank correlation coefficients between the evolutionary age (gene age and zinc finger divergence time) and gene expression abundance across early embryonic development, three directions of ESC differentiation in 4 mammals. **b** Heatmap shows spearman’s rank correlation coefficients between the evolutionary age (gene age and zinc finger divergence time) and gene expression abundance across organs from early organogenesis to adulthood and adult tissues or organs in 7 mammals. Samples with a correlation coefficient over 0.2 are listed in panel (**b**). For adult tissues or organs datasets of human and mouse, we select a dataset from each of the two species to display in the panel (**b**). The full raw data of this analysis are shown in Additional file 6. Six types of samples are marked in different colors, including early embryonic development (pink), endoderm differentiation (light blue), mesoderm differentiation (blue), ectoderm differentiation (purple), organs from early organogenesis to adulthood (brown) and adult tissues or organs (black)

In the preceding analysis, all kinds of expression level grades represent a wide range of abundances. In order to accurately analyze the relationship between intrinsic characteristics and expression level of genes, we made correlation analyses between their intrinsic characteristics (SDR, gene age and zinc finger divergence time) and gene expression abundance. As the results, gene age is positively correlated with gene expression abundance for PCGs in almost all samples (Fig. 5a & b, Additional file 6). However, this correlation for KZFPs was not significant in almost all samples (Fig. 5a & b, Additional file 6). As for the correlation between SDR value and expression abundance, there are weak correlations in a few samples (Additional file 6). More interestingly, there was a negative correlation between zinc finger divergence time and expression level of KZFPs in early embryonic development and early endoderm or mesoderm differentiation in human, rhesus, mouse and cattle (Fig. 5a), while there was a positive correlation between zinc finger divergence time and expression abundance of KZFPs in neuronal differentiation, and embryonic or adult tissues or organs (testis, brain, heart, etc.) (Fig. 5b, Additional file 6). In other words, KZFP genes encoding young zinc fingers tend to have higher expression level in early embryonic development and the ESC differentiation into endoderm or mesoderm, suggesting that young-zinc-finger-containing KZFPs may play important roles in these processes; so do the old-zinc-finger-containing KZFPs in their high-expression samples in mammals.

We further used hierarchical clustering method to analyze the normalized expression data of KZFPs in different samples, and found that the same or similar samples in different data sets could be preferentially clustered together (Fig. S4 in Additional file 1, zoom in for clear text, Additional file 7), indicating that our clustering analysis basically eliminated the batch differences among datasets. We found several conserved and interesting results (Figure S4 in Additional file 1, zoom in for clear text, Additional file 7): 1) part of young-zinc-finger-encoding KZFP genes are highly expressed in early embryonic development and reproductive organs (testis and ovary), such as ZNF479 and PRDM9 in human, respectively (Figure S5 in Additional file 1); 2) most of KZFP genes have high expression levels during the embryonic development of brain and kidney, except for several young-zinc-finger-encoding KZFP genes which are highly expressed in testis; 3) the overall expression level of most KZFP genes are relatively low in liver, and adult heart, kidney. These results revealed that although most of KZFP genes express widely across various spatiotemporal states from the qualitative viewpoint (Fig. 3 and Figure S3A in Additional file 1), there are differential expression patterns across different spatiotemporal states from the quantitative viewpoint (Figure S4 & S5 in Additional file 1, Additional file 7), suggesting the specific-high-expression KZFPs may play important roles in the corresponding states.

### The specific functions of the young or old-zinc-finger-containing KZFPs

**Fig. 6:**
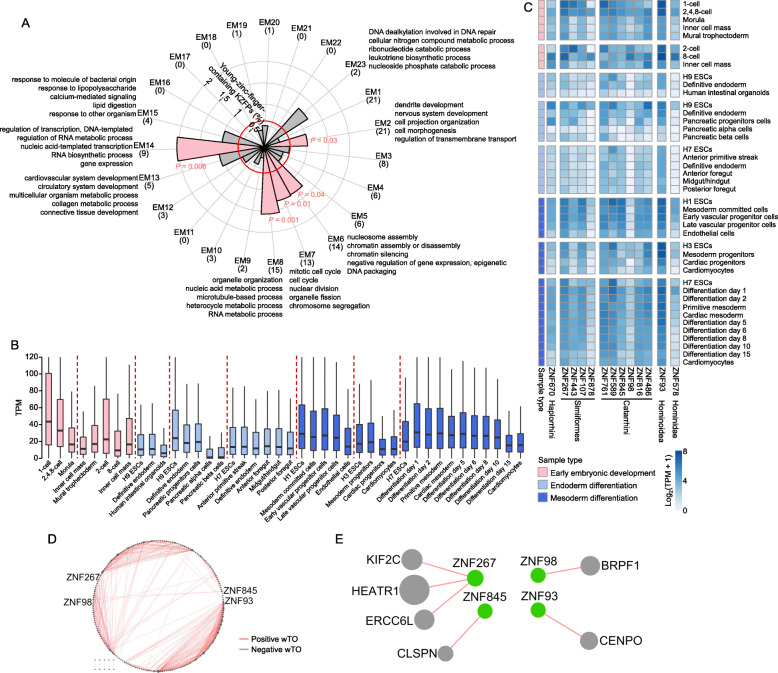
The expressional and functional characteristics of the co-expression modules containing young-zinc-finger-containing KZFPs. **a** Percentage of young-zinc-finger-containing KZFPs within each co-expression module. The genome-wide percentage of young-zinc-finger-containing KZFPs (0.61%) is indicated with a red circle. Modules exhibiting a significant excess (Bonferroni-corrected *P* < 0.05) of young-zinc-finger-containing KZFPs are indicated in pink, while nonsignificant modules are shown in gray. The corresponding corrected *P*-values are marked near the bars. For models in which the percentage of young-zinc-finger-containing KZFPs exceeds the genome-wide percentage, the significantly enriched GO terms are shown. The number of young-zinc-finger-containing KZFPs in each module is shown in brackets. **b** Boxplots show the TPM values of genes in EM7 during early embryonic development and the ESCs differentiation into endoderm or mesoderm. Samples in each dataset were sorted according to the time point of development or differentiation. Different datasets are separated by red dashed lines. **c** The expression abundance of young-zinc-finger-containing KZFPs in EM7 during early embryonic development and the ESCs differentiation into endoderm or mesoderm. The zinc finger divergence time of each KZFP is showed below the KZFP name. **d** Expression correlation as weighted topological overlap (wTO) between genes in EM7. Four young-zinc-finger-containing KZFPs (ZNF267, ZNF845, ZNF93 and ZNF98) are in green. **e** The wTO between four young-zinc-finger-containing KZFPs (ZNF267, ZNF845, ZNF93 and ZNF98) and other genes in EM7

Based on the conserved expression pattern described above, to further gain deep insights into the potential functions of young- or old-zinc-finger-containing KZFPs, the weighted gene co-expression network analysis (WGCNA) [26] was performed. Using human data, we identified 23 modules based on early development stages (EMs) (Fig. 6a) and 18 modules based on brain (forebrain and hindbrain) at various developmental stages from early organogenesis to adulthood (BMs) (Figure S6A in Additional file 1).

In total, 134 young-zinc-finger-containing KZFPs were involved in 17 out of the 23 EM modules, among which 8 modules contain more young-zinc-finger-containing KZFPs than the overall ratio. The representative enriched GO terms (biological process) for each module are shown for these 8 EMs (Fig. 6a). These enriched biological processes represent the general functions of young-zinc-finger-containing KZFPs in embryonic development and ESCs differentiation. Based on Fisher’s exact test, we found that young-zinc-finger-containing KZFPs are significantly enriched in EM2, EM6, 7, 8 and EM14. Further analysis of the expression pattern showed that among these five modules, the genes in EM7 were highly expressed in early development stages and early differentiation stages of ESCs, but decreased in late differentiation stage (Fig. 6b), indicating genes in EM7 may play important roles during the early stages of embryonic development and ESCs differentiation. Preceding results (Fig. 5) showed that young-zinc-finger-containing KZFPs tend to be expressed with high abundance during these stages. In EM7, there are 13 young-zinc-finger-containing KZFPs, including ZNF670 (Haplorrhini-specific), 4 Simiiformes-specific KZFPs (ZNF107, ZNF267, ZNF443, ZNF878), 6 Catarrhini-specific KZFPs (ZNF98, ZNF468, ZNF589, ZNF761, ZNF816, ZNF845), ZNF93 (Hominoidea-specific) and ZNF578 (Hominidae-specific). Their dynamic expression patterns are the same as the general trend of EM7 (Fig. 6c). Thus, we can infer their potential functions by deciphering the functional characteristics of EM7. GO term enrichment analysis revealed that genes in EM7 tend to participate in the biological processes related to cell cycle (Fig. 6a), which is a known process closely related to development and ESC differentiation [27–30]. Among them, ZNF589 is a known pluripotency maintaining protein through epigenetic repression of pro-differentiation genes [14].

In order to obtain the details about co-expression of genes in EM7, we calculated weighted topological overlap (wTO) [31] to obtain a signed co-expression network. After screening the credible co-expression gene pairs (see Methods), 206 genes were retained in EM7, including 4 young-zinc-finger-containing KZFPs (ZNF267, ZNF845, ZNF93 and ZNF98) (Fig. 6d, Additional file 8). Among them, ZNF267 have an positive correlation with kinesin family member 2C (KIF2C) (Fig. 6e), which was involved in cell cycle [32].

We also analyzed the interactors of these young-zinc-finger-containing KZFPs in EM7. Since the protein-protein interaction data we used was based on HEK293 cells [33] and HEK293T cells [34], we filtered the data to retain the genes expressed in at least 80% of the samples related to early development stages (see Methods). Three young-zinc-finger-containing KZFPs (ZNF267, ZNF578 and ZNF816) can interact with some early development related proteins (Figure S7A in Additional file 1, Additional file 9), such as ZNF267 interacts with ubiquitin protein ligase E3 component N-recognin 5 (UBR5), which is required for Wnt signal responses [35]. Overall, these results further show that young-zinc-finger-containing KZFPs in EM7 may play important roles in early embryonic development and ESC differentiation via participating in cell cycle related processes.

Among the 18 BMs, we found that BM5, with a high proportion of old-zinc-finger-containing KZFPs, tend to be involved in the functions closely related to brain development, such as nervous system development (Figure S6A in Additional file 1). The expression level of genes in BM5 are relatively high in post conception stages of forebrain and hindbrain, which are important periods of brain development (Figure S6B in Additional file 1). Three old-zinc-finger-containing KZFPs, including two KZFP genes encoding Mammalia-specific zinc fingers (ZNF205, ZNF436), and ZNF764 with Theria-specific zinc fingers, in BM5 also have relatively high expression level in post conception stage forebrain and hindbrain (Figure S6C in Additional file 1). We next obtained 425 genes with credible co-expression pairs in BM5 (Figure S6D in Additional file 1, Additional file 8), including ZNF436 and ZNF764. ZNF764 is positively correlated with several genes involved in brain development (Figure S6E in Additional file 1), such as platelet activating factor acetylhydrolase 1b catalytic subunit 3 (PAFAH1B3) [36]. After screening the genes expressed in at least 80% samples in brain development, we analyzed the interactors of old-zinc-finger-containing KZFPs in BM5, and found that ZNF764 interacts with protein arginine methyltransferase 1 (PRMT1) (Figure S7B in Additional file 1, Additional file 9), which is involved in brain development [37]. These results suggest that old-zinc-finger-containing KZFPs in BM5 may participate in brain development.

## Discussion

Since the completion of the human genome project, it has been found that hundreds of C2H2 zinc finger proteins contain a KRAB domain [1, 3, 38]. Compared with other species, it is found that this large family experienced rapid expansion in a short period of evolution, and the species specificity is very strong [1, 5, 7, 18]. Therefore, what kind of biological function does such a large and rapidly evolving family have is one of the fundamental questions in this field. However, the functions of many KZFPs are still unknown yet, which makes it difficult to understand the functional characteristics of KZFPs with different evolutionary ages. Since the expression and structural characteristics could provide important clues of protein function, to systematically understand the functional characteristics of this family and their relevant with the rapid evolution of this family, it is essential to explore what structural and expressional features belonging to the KZFP family members emerging in different evolutionary nodes. In this study, we comprehensively analyzed the characteristics of structure, expression of KZFPs and explored the relationships between them and evolutionary age grades. Surprisingly, we found several conserved paradoxical relationships as follows.

Firstly, young domains usually tend to be disordered, while KRAB domains as young domains, tend to be completely structured in 64 species. Since KRAB domains mainly contribute to the protein-protein interactions with other transcriptional co-regulators [3, 23], the completely structured KRAB domains may lead to a kind of monotonous and unchangeable regulatory pattern of KZFPs, which maybe one of the important guarantees to maintain the stability of common KRAB’n’KAP1 system [39].

Interestingly, young proteins tent to be disordered, but most KZFPs (about 90% of the total KZFPs) in all gene age grade are highly structured; as exceptions, old-zinc-finger-containing KZFPs (about 10% of the total KZFPs) have relatively disordered KRAB domains and linker regions. The conformation of highly structured proteins and domains usually are rigid [10], suggesting the functional mechanism of these proteins or domains tends to be monotonous and unchangeable. Therefore, these results suggested that, in general, most KRAB domains and KZFPs are rigid and not easy to change its conformation. Such structural characteristics makes most KZFPs, except for almost all old-zinc-finger-containing KZFPs, share KAP1-related functions by having a strong recruitment strength of KAP-1 [34], which act as a scaffold for other histone-modifying and -binding factors, to compose a transcriptional regulating complex [40, 41]. For example, ZNF90 (Figure S8A & S8B in Additional file 1) and ZNF287 (Figure S8C & S8D in Additional file 1) are two KZFPs containing young and mid-age zinc fingers respectively. Both of them have a completely structured KRAB domain and linker region (SDR = 0, Figure S8A & S8C in Additional file 1) and they tend to interact with KAP1 and KAP1-associated proteins (Figure S8B, D in Additional file 1) [34]. On the other side, disordered proteins and domains lack stable three-dimensional structures [10], but can perform important diverse functions, such as chaperones [24]. Thus, the KZFPs containing old zinc fingers have relatively disordered KRAB domains, and these KZFPs can play a variety of functions by relatively disordered KRAB domains interacting with multiple types of proteins [34], responsible for atypical, distinct features hinting at diverse roles. For example, ZKSCAN3 (Figure S8E & S8F in Additional file 1) is an old-zinc-finger-containing KZFP. Its KRAB domain and linker region are highly disordered (SDR: 59, 47.3% respectively, Figure S8E in Additional file 1), and it can interact with various other proteins besides KAP-1 (Figure S8F in Additional file 1) [34].

Secondly, young genes, especially those encoding highly structured proteins, are generally expressed with a spatiotemporal-specific pattern [20–23], however, KZFP genes tend to be ubiquitously expressed regardless of the gene age, zinc finger divergence time and protein disorder degree in mammals. Meanwhile, young genes tend to be expressed with a low abundance, whereas KZFP genes tend to be with a medium abundance. In view of the extensive requirement to repress transposable elements (TEs) in a wide range of biological processes, including but not limited to ESCs [42, 43], embryonic development [44, 45] and adult cells or tissues [46, 47], this requirement maybe one of the driving forces for ubiquitous and medium-abundance expression in KZFP family. Besides, KZFPs also have many other functions [3, 48, 49], such as cell differentiation [50–54], metabolism [55–58], genomic imprinting [48–50] and meiotic recombination [51, 52]. Thus, a wide range of functional requirements besides repressing TEs may also need the special expression pattern of KZFPs, and may be a driver of their rapid expansion during evolution. Additionally, KZFP genes encoding young zinc fingers tend to have higher expression level in early embryonic development and the differentiation from ESCs to endoderm or mesoderm in mammals, and KZFP genes encoding old zinc fingers tend to have higher expression level in the embryonic or adult brain and other organs (testis, heart, etc.) across some mammals. More specifically, the overall expression level of most KZFP genes are relatively low in liver, and adult heart, kidney; part of young-zinc-finger-encoding KZFP genes are highly expressed in early embryonic development and reproductive organs (testis and ovary), and most of KZFP genes have high expression levels during the embryonic development of brain and kidney, except for several young-zinc-finger-encoding KZFP genes which are highly expressed in testis. These results indicate that KZFP family has special and conserved structural and expressional features in mammals.

Furthermore, KZFPs containing young zinc fingers are preferentially recruited into functions related to early development-related processes, suggesting that young-zinc-finger-containing KZFPs (*e. g*. ZNF267) are specifically recruited into embryonic development and the differentiation from ESCs to endoderm or mesoderm, such as ZFP809 silencing endogenous retroelements in ESCs and embryonic development [42, 44], ZNF114 and ZNF589 as known pluripotency maintaining proteins through epigenetic repression of pro-differentiation genes [14], and ZNF611 along with several evolutionary recent KZFPs taming the activity of enhancers embedded in young TEs during human early embryogenesis [43]. On the other hand, KZFP genes encoding old zinc fingers (*e. g.* ZNF764) tend to participate in functions related to brain development. These KZFPs may also inhibit TEs by interacting with KAP1 in brain [47, 59, 60]. Besides inhibiting TEs, the expression of some KZFPs containing old zinc fingers (such as ZNF202) shows correlation with the expression of their target genes inferred based on ChIP-exo or ChIP-seq data in the developing human brain [16]. Considering that old-zinc-finger-containing KZFPs can play diverse functions [34], for example genome architecture or RNA processing, due to containing relatively disordered KRAB domains and linker regions, these KZFPs may play more important roles in brain development besides repressing TEs.

## Conclusion

Based on the results obtained from this study, we can conclude that KZFP family has evolutionarily conserved and special features in structure and expression. The special characteristics of KZFP family discovered in this study show a novel understanding of the conserved relationship between gene intrinsic properties and molecular phenotypic features outside the generalized knowledge in this field, and provide valuable clues for the further detailed functional study of this amazing large family.

## Methods

### The identification of protein domains in 64 species

The protein sequences of 64 species from 64 genera across the superclass of Sarcopterygii (Additional file 2) were downloaded from Ensembl database [61], and the HMM files of all protein domains were download from Pfam v29.0 [62]. All domains in proteins were identified using HMMER v3.1b2 [63] with both protein E value < 0.01 and domian E value < 0.01. The domain age was defined by the oldest taxon in which the protein domain first appeared in the Pfam species tree [20, 21]. The construction of species tree was based on NCBI taxonomy (https://www.ncbi.nlm.nih.gov/taxonomy/). Evolutionary distance between species were estimated by TimeTree [64].

### The definition of the gene ages and zinc finger divergence times of KZFPs

The proteins containing both a KRAB domain and C2H2 zinc fingers were defined as KZFPs. The gene ages of all PCGs used in this study were defined by a consensus gene age dataset which integrated 13 orthology inference algorithms [65]. For the 7 mammals in this study, Mammalia-specific genes are regarded as the young genes; The genes whose specificity are among the grades from Eumetazoa to Vertebrata are regarded as the mid-age genes; others are regarded as old genes. The zinc finger divergence times of KZFPs were inferred according to the similarity between the key amino acids in zinc fingers. The detailed method for that is the same as a previous study [5]. According to zinc finger divergence time, KZFPs are also classified into 3 age grades (Additional file 10).

### The SDR of proteins and domains

The longest protein encoded by each gene was selected as the representative protein for subsequent analyses. SPOT-Disorder-Single [66] were used to obtain the disorder score of each amino acid in a protein or domain. The disorder rate of a protein is the ratio of the number of disordered amino acids (the amino acid is identified as ‘D’ (disordered)) to the total number of amino acids.

### RNA-Seq data collection

The RNA-Seq data was downloaded from GEO and ArrayExpress database: human early embryonic development (GSE72379, GSE101571); differentiation of hESCs into endoderm (E-MTAB-3158, GSE44875, GSE52657); differentiation of hESCs into mesoderm (GSE54968, GSE74665, GSE76523); differentiation of hESCs into ectoderm (GSE56152, GSE56796, GSE80264); organs at developmental stages from early organogenesis to adulthood in human (E-MTAB-6814); human adult tissues/organs (E-MTAB-2836, Genotype-Tissue Expression (GTEx) (https://www.gtexportal.org/home/)); chimpanzee adult organs (GSE69241); rhesus early embryonic development (GSE103313); organs at developmental stages from early organogenesis to adulthood in rhesus (E-MTAB-6813); rhesus adult organs (GSE69241); mouse early embryonic development (GSE70605, GSE98150); differentiation of mESCs into endoderm (GSE92572); differentiation of mESCs into mesoderm (GSE47948); differentiation of mESCs into ectoderm (GSE94364); organs at developmental stages from early organogenesis to adulthood in mouse (E-MTAB-6798); mouse adult tissues/organs (GSE36025, GSE41637); organs at developmental stages from early organogenesis to adulthood in rat (E-MTAB-6811); cattle early embryonic development (GSE143848); cattle adult organs (GSE41637); organs at developmental stages from early organogenesis to adulthood in opossum (E-MTAB-6833).

### RNA-Seq data processing

FastQC v 0.11.7 [67] and trimmomatic v 0.39 [68] were used for read trimming and filtering. The clean reads were mapped to the human genome build GRCh38 (hg38) using Salmon v 0.11.0 [69]. The transcripts per kilobase of exon model per million mapped reads (TPMs) and read counts of genes were calculated using Salmon v 0.11.0 [69] and tximport [70]. Genes with read counts over 10 are considered to be expressed. For each dataset, we used the upper and lower quartiles of TPMs of all expressed genes to divide them three expression levels: low-abundant level (L), the genes with TPMs lower than lower quartile; medium-abundant level (M). the genes with TPMs between the lower quartile and the upper quartile; high-abundant level (H), the genes with TPMs higher than the upper quartile.

### Cluster analysis

We first used the ComBat function based on an empirical Bayesian framework [71] in R package SVA [72, 73] to remove the batch effect between different datasets. Then, z-score was used to standardize the expression value. The hierarchical clustering method was used to analyze the normalized expression data of KZFPs in different samples of each species.

### Protein-protein interaction network

All interactors of 139 KZFPs (Additional file 9) were obtained from Hughes’s data [33] and Trono’s data [34] detected by affinity purification and mass spectrometry (AP-MS). Known KAP1 complex proteins (SIRT1, SMARCAD1, HP1α, and HP1γ) and preys that only appear in interactomes alongside KAP1 were marked as KAP1-associated proteins [34]. The KZFP interaction network was built using Cytoscape v 3.4.0 [74–76].

### Co-expression network analysis

We selected the samples related to early development stages, including human early embryonic development, differentiation of hESCs into endoderm and mesoderm as an early development dataset. And we chose brain (forebrain and hindbrain) at developmental stages from early organogenesis to adulthood in human as a dataset related to the middle and late development stages. For each dataset, TPM values were log_2_ transformed after adding a pseudo-count of 1 to avoid log transforming zero, then these transformed TPM values were used as input. We required genes to be expressed in at least one sample [77] (read count > 10) and with a CV (variance/mean) over 0.08, which were considered to have sufficient information according to generally WGCNA practices [77, 78]. R pakeage WGCNA [26] was used to constructed coexpression modules based on the two datasets, respectively. We used the powerEstimation function to get the optimal fit in early development dataset (best power is 12, Figure S9A in Additional file 1) and brain development dataset (best power is 12, Figure S9B in Additional file 1). To identify more modules with a decent module size, we set the deep split parameter to 4 and minimum module size to 150 in the blockwiseModules function, respectively.

### Weighted topological overlap (wTO)

The wTO is used to estimate how a set of genes of interest is correlated. The higher the absolute value of wTO, the stronger the positive or negative correlation between the two genes. The R package wTO [31] was used to calculate the wTO of the genes in the co-expression modules of EM7 and BM5 in this study. The parameters were set to Pearson’s product moment correlation coefficient and 1000 bootstraps resampling [79, 80]. The final results were filtered according to two parameters: a probability of 0.10 for having random wTO based on empirical quantile, and *P* value adjusted by Benjamini-Hochberg method < 0.001 [31, 79, 80]. The co-expression network was built using Cytoscape v 3.4.0 [74–76].

### The over- or under-representation analysis

This was performed using the method as previously described [20]. Briefly, to analyse the over- or under-representation strengths of genes in each class relative to the background, we used the method based on hypergeometric distribution. *P* values were corrected by Bonferroni correction. The over- or under-representation strengths of each class were represented by -log (*p*) or log (*p*).

For GO term enrichment analysis, we first removed genes without GO term annotations. Subsequently, we retained the genes expressed in at least one sample as the background.

## Supplementary Information

**Additional file 1: Figure S1.** The schematic diagram of the domain architecture of KZFP and the key amino acids in zinc finger binding to DNA. **Figure S2.** The SDR values of KRAB domains with different gene age grades in 7 mammals. **Figure S3.** The expression pattern of KZFP genes with different zinc finger divergence time grades. Figure S4. The expression pattern of KZFP genes in 7 mammals. **Figure S5.** The highly expressed KZFP genes in human. **Figure S6.** The expressional and functional characteristics of the co-expression modules containing old-zinc-finger-containing KZFPs. **Figure S7.** The PPIs of young- or old-zinc-finger-containing KZFPs. **Figure S8.** The SDR values and interactors of ZKSCAN3, ZNF287 and ZNF90. **Figure S9.** The WGCNA parameter for early development dataset (A) and brain development dataset (B).

**Additional file 2.** Percentage of each disorder grade of KRAB domains and other chordata-specific domains in each of the 64 species (related to Fig. 1).

**Additional file 3.** The annotation information and SDR values of KZFPs in 7 mammals (related to Fig. 2 and Figure S2 in Additional file 1).

**Additional file 4.** The annotation information and SDR values of PCGs in 7 mammals (related to Fig. 2 and Figure S2 in Additional file 1).

**Additional file 5.** The number and percentage of expressed KZFPs (related to Figs. 3 and 4 and Figure S3 in Additional file 1).

**Additional file 6.** The spearman’s rank correlation coefficients between gene intrinsic properties (gene age, zinc finger divergence time or SDR values) and expression level (TPM) in 7 mammals (related to Fig. 5).

**Additional file 7.** The expression z-score of KZFPs in 7 representative mammals (related to Figure S4 & S5 in Additional file 1).

**Additional file 8.** The wTO of genes in EM7 or BM5 (related to Fig. 6 and Figure S6 in Additional file 1).

**Additional file 9.** The protein-protein interaction network of human KZFPs and the expression of baits and preys in each sample.

**Additional file 10.** The number of PCGs and KZFP genes in each evolutionary age grade in 7 mammals.

## Data Availability

The databases used in this study are as follows. All databases are open to public access. Ensembl database (http://asia.ensembl.org/index.html) [61], Pfam database (http://pfam.xfam.org/) [62], NCBI taxonomy (https://www.ncbi.nlm.nih.gov/taxonomy/), TimeTree (http://timetree.org/) [64], the consensus gene age dataset [65], and the zinc finger divergence times of KZFPs were inferred from a published paper [5]. ArrayExpress database (https://www.ebi.ac.uk/arrayexpress/): E-MTAB-2836, E-MTAB-3158, E-MTAB-6798, E-MTAB-6811, E-MTAB-6813, E-MTAB-6814, E-MTAB-6833. GEO database (https://www.ncbi.nlm.nih.gov/geo/): GSE101571, GSE103313, GSE143848, GSE36025, GSE41637, GSE44875, GSE47948, GSE52657, GSE54968, GSE56152, GSE56796, GSE69241, GSE70605, GSE72379, GSE74665, GSE76523, GSE80264, GSE92572, GSE94364, GSE98150. RNA-seq data from Genotype-Tissue Expression (GTEx (https://www.gtexportal.org/home/). Protein-protein interaction network: Hughes’s data [33] and Trono’s data [34].

## Abbreviations

KZFP: KRAB domain-containing zinc finger protein
ESCs: Embryonic stem cells
SDR: Structural disordered ratio
PCGs: Protein-coding genes
L: Low-abundant level
M: Medium-abundant level
H: High-abundant level
WGCNA: Weighted gene co-expression network analysis
EMs: Modules based on early development stages
BMs: Modules based on brain (forebrain and hindbrain) at various developmental stages from early organogenesis to adulthood
wTO: Weighted topological overlap
KIF2C: Kinesin family member 2C
UBR5: Ubiquitin protein ligase E3 component N-recognin 5
PRMT1: Protein arginine methyltransferase 1
TPMs: Transcripts per kilobase of exon model per million mapped reads
AP-MS: Affinity purification and mass spectrometry
