# Supplementary material for: Conserved paradoxical relationships among the evolutionary, structural and expressional features of KRAB zinc-finger proteins reveal their special functional characteristics
Source: BMC Mol Cell Biol. 2021 Jan 22;22:7. doi: 10.1186/s12860-021-00346-w (PMC7821633; doi:10.1186/s12860-021-00346-w)
Supplement: Supplementary file 1 — Additional file 1: Figure S1. The schematic diagram of the domain architecture of KZFP and the key amino acids in zinc finger binding to DNA. Figure S2. The SDR values of KRAB domains with different gene age grades in 7 mammals. Figure S3. The expression pattern of KZFP genes with different zinc finger divergence time grades. Figure S4. The expression pattern of KZFP genes in 7 mammals. Figure S5. The highly expressed KZFP genes in human. Figure S6. The expressional and functional characteristics of the co-expression modules containing old-zinc-finger-containing KZFPs. Figure S7. The PPIs of young- or old-zinc-finger-containing KZFPs. Figure S8. The SDR values and interactors of ZKSCAN3, ZNF287 and ZNF90. Figure S9. The WGCNA parameter for early development dataset (A) and brain development dataset (B). [file 12860_2021_346_MOESM1_ESM.pdf]

Supplementary figure legends

Figure S1. The schematic diagram of the domain architecture of KZFP and the key amino acids in zinc finger binding to DNA.

Figure S2. The SDR values of KRAB domains with different gene age grades in 7 mammals. *(A-D)* Box-plots of SDR values of KRAB domain *(A)*, C2H2 zinc fingers *(B)*, other domains *(C)* and linker region *(D)* in KZFPs with different gene age grades in 7 mammals. *(E&F)* Comparison of SDR values of KZFPs *(E)* and PCGs *(F)* with different gene age grades in 7 mammals. For box-plots, the values of upper and lower quartiles are indicated as upper and lower edges of the box, and the median values of median are indicated as a bar in the box. The number of KZFPs and PCGs in each category is in Additional file 10. The category with gene number 0 is displayed in gray. The differences of SDR value between different categories are examined by Mann–Whitney U test. The corrected P values are shown in the top of each panel. ns: $p > 0.05$, *: $p < 0.05$, **: $p < 0.01$, ***: $p < 0.001$, ****: $p < 0.0001$. Gene age grade: CO-Op, cellular organisms - opisthokonta; Eu-Ve, eumetazoa - vertebrata; Ma, mammalia.

Figure S3. The expression pattern of KZFP genes with different zinc finger divergence time grades. Samples in 7 mammals, including early embryonic development, three directions of ESC differentiation, organs from early organogenesis to adulthood and adult tissues or organs were used in the analysis. *(A)* Expression width of KZFP genes with three zinc finger divergence grades. The differences of expression width between young-zinc-finger-containing KZFPs and KZFPs with old or mid zinc finger divergence time grades are examined by Mann–Whitney U test. The corrected P values are shown in the top of each panel. ns: $p > 0.05$, *: $p < 0.05$, **: $p < 0.01$, ***: $p < 0.001$, ****: $p < 0.0001$. *(B)* The proportion of three expression level grades of KZFPs with each zinc finger divergence time grade. The over/under representation strength shows KZFP genes with different expression level grades relative to all KZFPs in each zinc finger divergence time grade in each sample. Over representation strength: +, $P \geq 0.05$; ++, $P < 0.05$; +++, $P < 10^{-10}$. Under representation strength: −, $P \geq 0.05$; −−, $P < 0.05$; −−−, $P < 10^{-10}$. The number in the box on the left represents the total number of samples. The number of KZFPs in each category is in Additional file 10.

Figure S4. The expression pattern of KZFP genes in 7 mammals. Heatmaps show z-score normalized expression data for KZFPs in human *(A)*, chimpanzee *(B)*, rhesus *(C)*, mouse *(D)*, rat *(E)*, cattle *(F)* and opossum *(G)*. Hierarchical clustering method was used in both samples and KZFP genes. The full expression z-scores of each KZFP are shown in Additional file 6.

Figure S5. The highly expressed KZFP genes in human. KZFP genes with z-score over 5 are defined as highly expressed KZFP genes. Samples with at least one highly expressed KZFP genes are shown. KZFPs containing young zinc fingers are in green.

Figure S6. The expressional and functional characteristics of the co-expression modules containing old-zinc-finger-containing KZFPs. *(A)* Percentage of old-zinc-finger-containing KZFPs within each coexpression module. The genome-wide percentage of old-zinc-finger-containing KZFPs (0.15 %) is indicated with a red line. Modules exhibiting a significant excess (Bonferroni-corrected P < 0.05) of old-zinc-finger-containing KZFPs are indicated in pink, while nonsignificant modules are shown in gray. The corresponding corrected P-values are marked near the bars. For models in which the percentage of old-zinc-finger-containing KZFPs exceeds the genome-wide percentage, the significantly enriched GO terms are shown. The number of old-zinc-finger-containing KZFPs in each module is shown in brackets. *(B)* Boxplots show the TPM values of genes in BM5 in developmental stages of brain (forebrain and hindbrain) from early organogenesis to adulthood in human. Samples in each organ were sorted according to the time point of development from early to late. Different organ are separated by red dashed lines. *(C)* The expression abundance of old-zinc-finger-containing KZFPs in PM6 in different brain-related samples. The zinc finger divergence time of each KZFP is marked below the KZFP name. *(D)* Expression correlation as weighted topological overlap (wTO) between genes in BM5. Two old-zinc-finger-containing KZFPs (ZNF436 and ZNF764) are in green. *(E)* The wTO between two old-zinc-finger-containing KZFPs (ZNF436 and ZNF764) and other genes in BM5. The genes associated with brain development is in purple.

Figure S7. The PPIs of young- or old-zinc-finger-containing KZFPs. *(A)* The PPI network of three young-zinc-finger-containing KZFPs (ZNF267, ZNF587, ZNF816) in EM7. *(B)* The PPIs of ZNF764, an old-zinc-

finger-containing KZFP in BM5. The genes related to embryonic development or ESC differentiation are in purple *(A)*; and PRMT1, related to brain development, is in orange *(B)*. KAP1 or KAP1-associated proteins are in diamond box, and non-KAP1-associated proteins are in elliptical frame.

Figure S8. The SDR values and interactors of ZKSCAN3, ZNF287 and ZNF90. *(A-C)* The SDR value of ZNF90 *(A)*, ZNF287 *(B)* and ZKSCAN3 *(C)* at each amino acid position. *(D-F)* the interactors of ZNF90 *(D)*, ZNF287 *(E)* and ZKSCAN3 *(F)*. KAP1 or KAP1-associated proteins are in yellow, and non-KAP1-associated proteins are in blue.

Figure S9. The WGCNA parameter for early development dataset (A) and brain development dataset (B). We used the results of the pickSoftThreshold function in WGCNA to generate these plots. The red line marks the R^2 of 0.9.

Figure S1

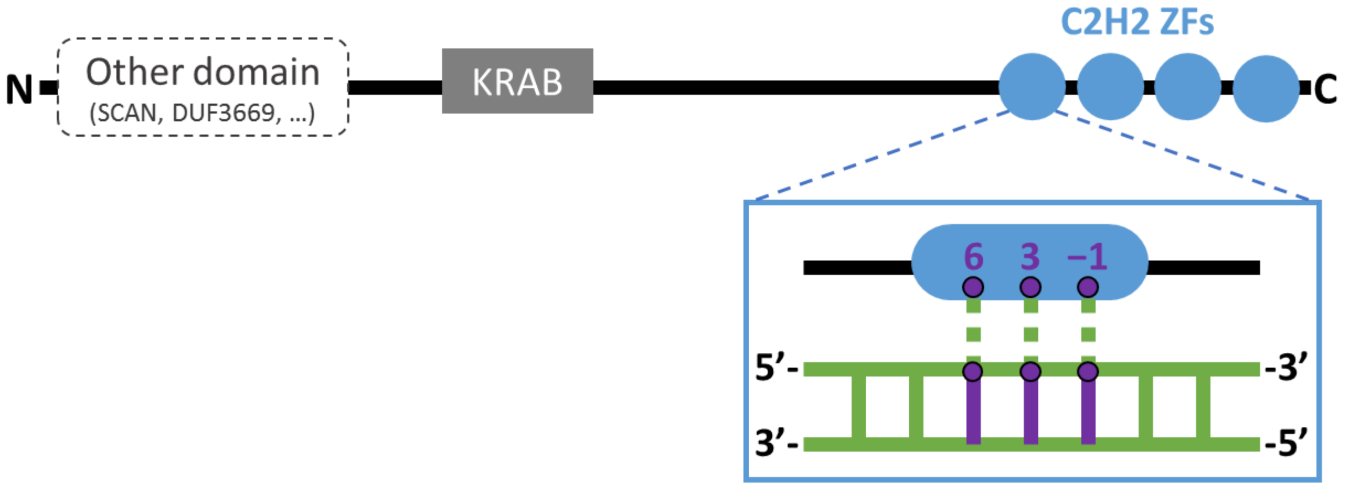

Figure S2

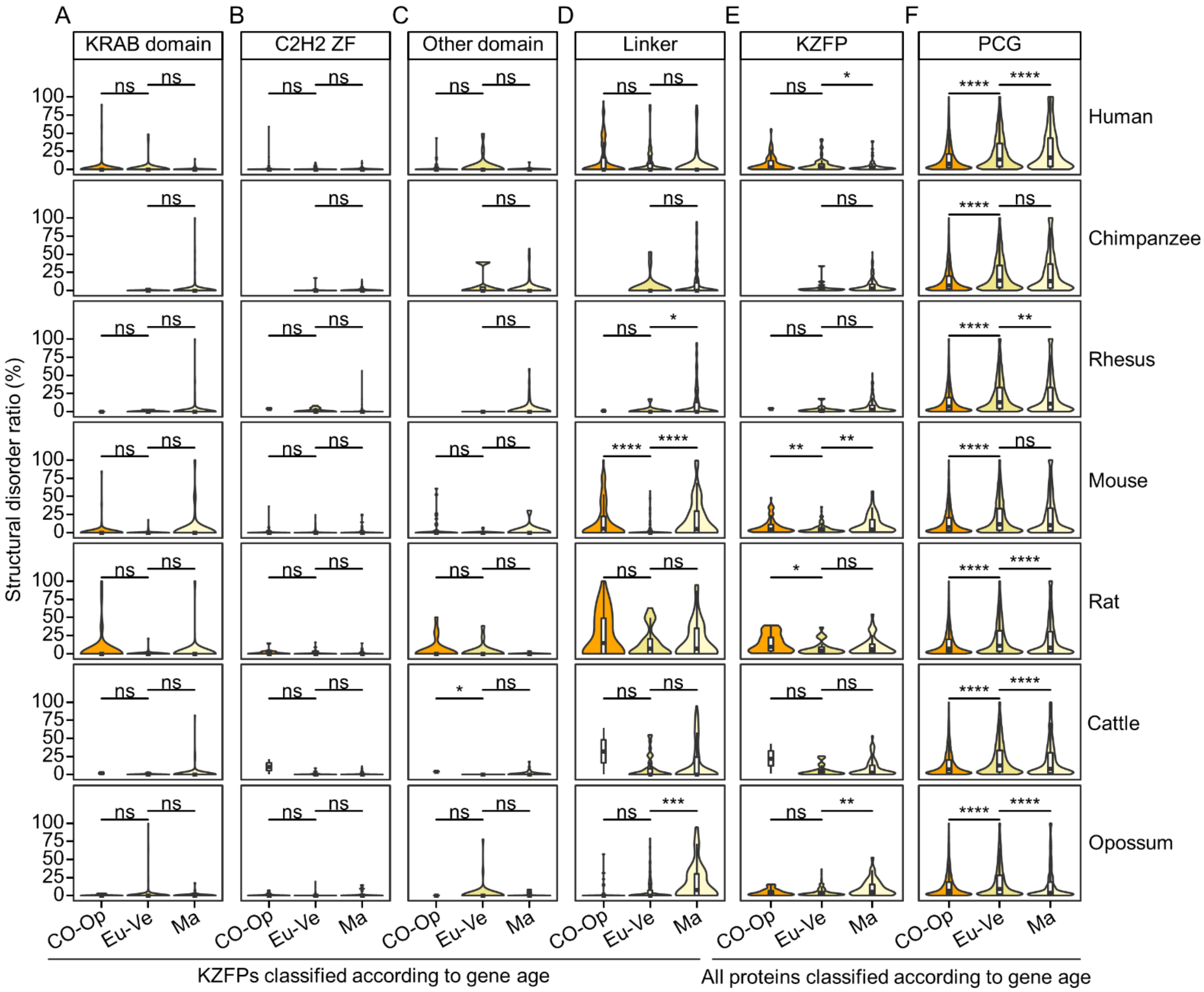
A
B
C
D
E
F
KRAB domain
C2H2 ZF
Other domain
Linker
KZFP
PCG
Human
Chimpanzee
Rhesus
Mouse
Rat
Cattle
Opossum
Structural disorder ratio (%)
100
75
50
25
0
ns
*
**
***
****
CO-Op
Eu-Ve
Ma
KZFPs classified according to gene age
All proteins classified according to gene age

Figure S3

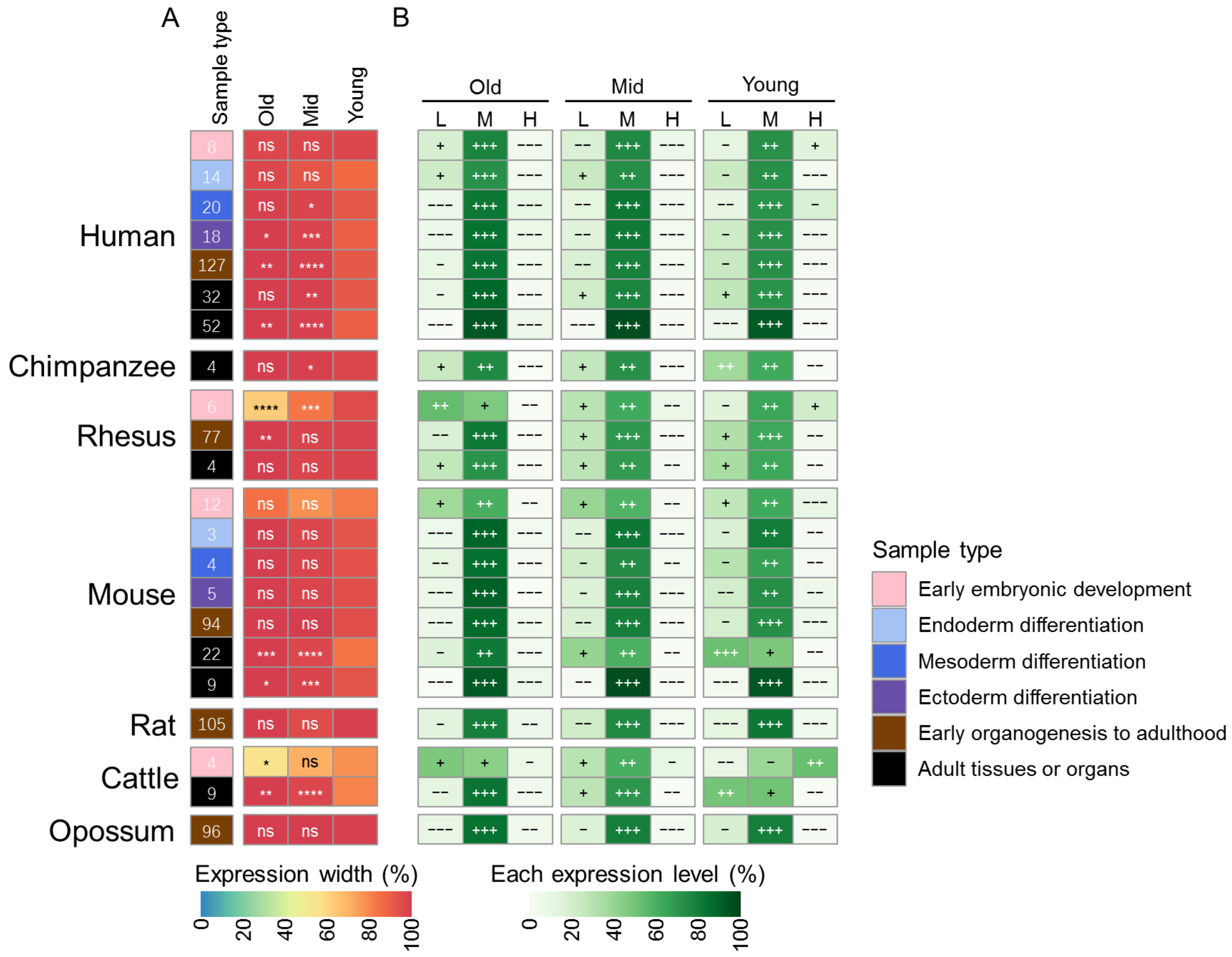

A
B
Sample type
Old
Mid
Young
L
M
H
Human
Chimpanzee
Rhesus
Mouse
Rat
Cattle
Opossum
Expression width (%)
Each expression level (%)
Early embryonic development
Endoderm differentiation
Mesoderm differentiation
Ectoderm differentiation
Early organogenesis to adulthood
Adult tissues or organs

Figure S4A

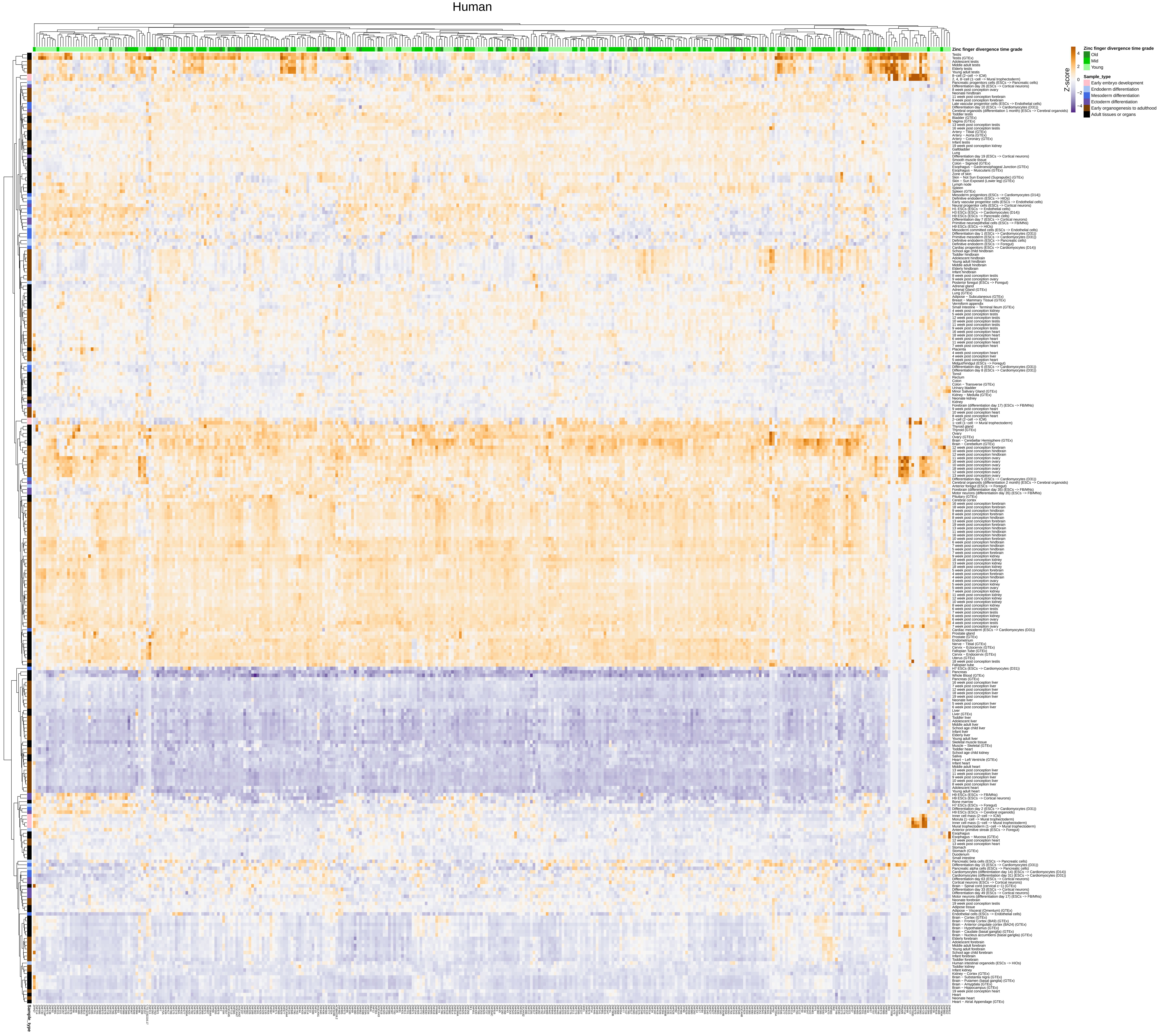
Human
Zinc finger divergence time grade
Old
Mid
Young
Z-score
Sample_type
Early embryo development
Endoderm differentiation
Mesoderm differentiation
Ectoderm differentiation
Early organogenesis to adulthood
Adult tissues or organs

Figure S4B

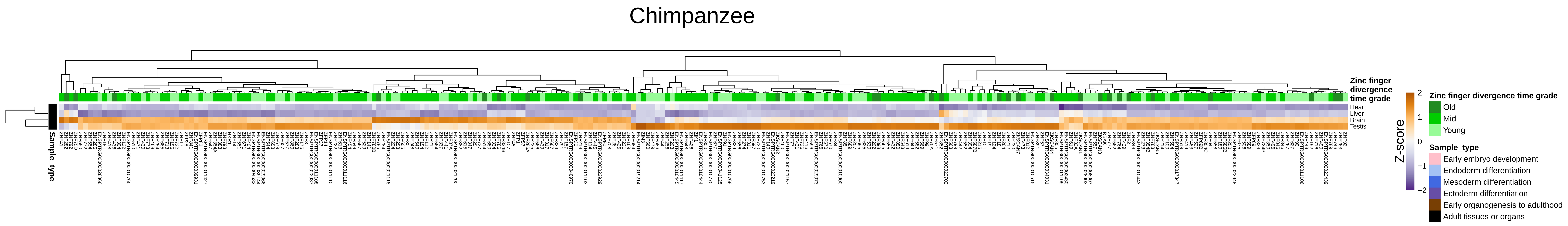

Chimpanzee
Zinc finger divergence time grade
Heart
Liver
Brain
Testis
Sample_type
Z-score
2
1
0
−1
−2
Zinc finger divergence time grade
Old
Mid
Young
Sample_type
Early embryo development
Endoderm differentiation
Mesoderm differentiation
Ectoderm differentiation
Early organogenesis to adulthood
Adult tissues or organs

Figure S4C

Rhesus

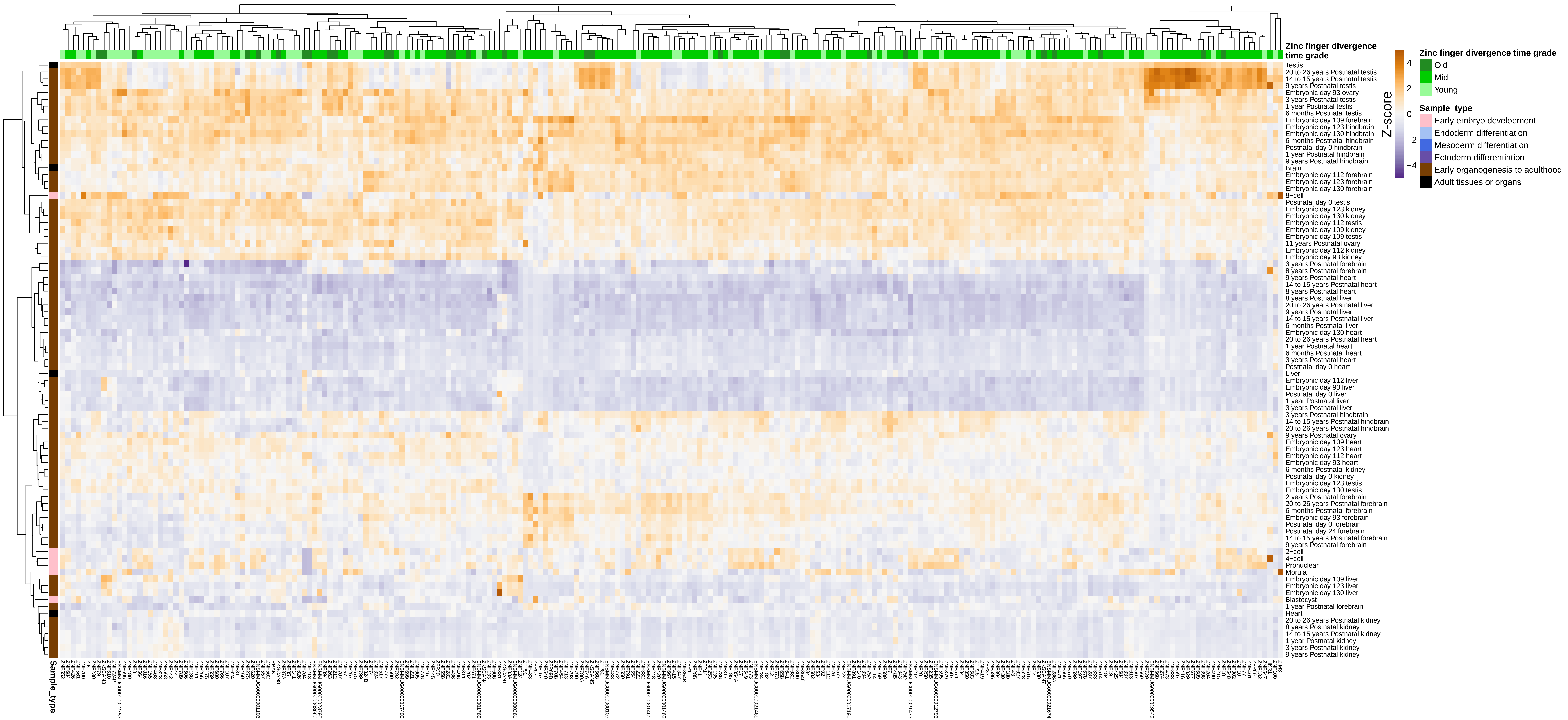

Figure S4D

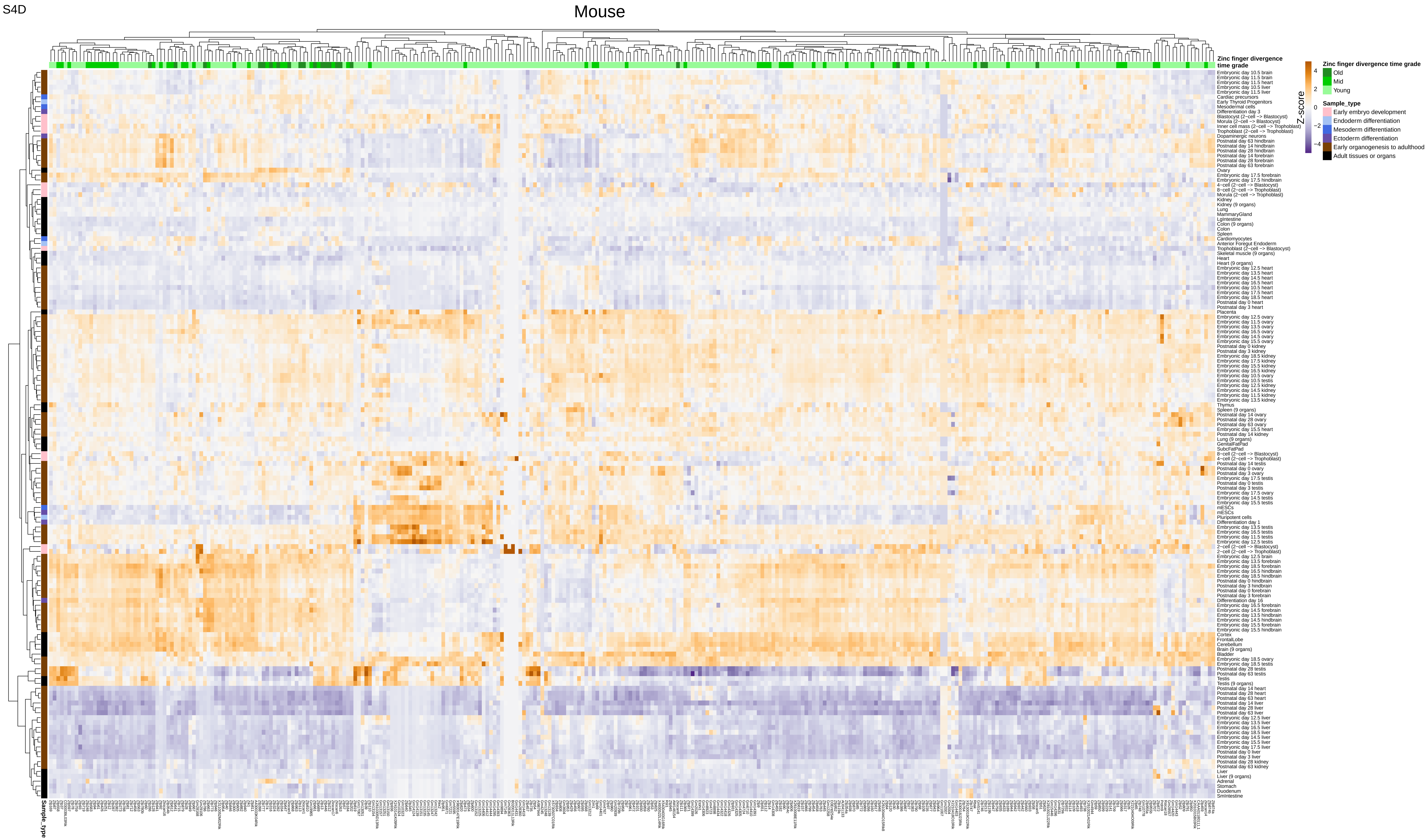

Mouse
Zinc finger divergence time grade
Embryonic day 10.5 brain
Embryonic day 11.5 brain
Embryonic day 11.5 heart
Embryonic day 10.5 liver
Embryonic day 11.5 liver
Cardiac precursors
Early Thyroid Progenitors
Mesodermal cells
Differentiation day 3
Blastocyst (2−cell −> Blastocyst)
Morula (2−cell −> Blastocyst)
Inner cell mass (2−cell −> Trophoblast)
Trophoblast (2−cell −> Trophoblast)
Dopaminergic neurons
Postnatal day 63 hindbrain
Postnatal day 14 hindbrain
Postnatal day 28 hindbrain
Postnatal day 14 forebrain
Postnatal day 28 forebrain
Postnatal day 63 forebrain
Ovary
Embryonic day 17.5 forebrain
Embryonic day 17.5 hindbrain
4−cell (2−cell −> Blastocyst)
8−cell (2−cell −> Trophoblast)
Morula (2−cell −> Trophoblast)
Kidney
Kidney (9 organs)
Lung
MammaryGland
LgIntestine
Colon (9 organs)
Colon
Spleen
Cardiomyocytes
Anterior Foregut Endoderm
Trophoblast (2−cell −> Blastocyst)
Skeletal muscle (9 organs)
Heart
Heart (9 organs)
Embryonic day 12.5 heart
Embryonic day 13.5 heart
Embryonic day 14.5 heart
Embryonic day 16.5 heart
Embryonic day 10.5 heart
Embryonic day 17.5 heart
Embryonic day 18.5 heart
Postnatal day 0 heart
Postnatal day 3 heart
Placenta
Embryonic day 12.5 ovary
Embryonic day 11.5 ovary
Embryonic day 13.5 ovary
Embryonic day 16.5 ovary
Embryonic day 14.5 ovary
Embryonic day 15.5 ovary
Postnatal day 0 kidney
Postnatal day 3 kidney
Embryonic day 18.5 kidney
Embryonic day 17.5 kidney
Embryonic day 15.5 kidney
Embryonic day 16.5 kidney
Embryonic day 10.5 ovary
Embryonic day 10.5 testis
Embryonic day 12.5 kidney
Embryonic day 14.5 kidney
Embryonic day 11.5 kidney
Embryonic day 13.5 kidney
Thymus
Spleen (9 organs)
Postnatal day 14 ovary
Postnatal day 28 ovary
Postnatal day 63 ovary
Embryonic day 15.5 heart
Postnatal day 14 kidney
Lung (9 organs)
GenitalFatPad
SubcFatPad
8−cell (2−cell −> Blastocyst)
4−cell (2−cell −> Trophoblast)
Postnatal day 14 testis
Postnatal day 0 ovary
Postnatal day 3 ovary
Embryonic day 17.5 testis
Postnatal day 0 testis
Postnatal day 3 testis
Embryonic day 17.5 ovary
Embryonic day 14.5 testis
Embryonic day 15.5 testis
mESCs
mESCs
Pluripotent cells
Differentiation day 1
Embryonic day 13.5 testis
Embryonic day 16.5 testis
Embryonic day 11.5 testis
Embryonic day 12.5 testis
2−cell (2−cell −> Blastocyst)
2−cell (2−cell −> Trophoblast)
Embryonic day 12.5 brain
Embryonic day 13.5 forebrain
Embryonic day 18.5 forebrain
Embryonic day 16.5 hindbrain
Embryonic day 18.5 hindbrain
Postnatal day 0 hindbrain
Postnatal day 3 hindbrain
Postnatal day 0 forebrain
Postnatal day 3 forebrain
Differentiation day 16
Embryonic day 16.5 forebrain
Embryonic day 14.5 forebrain
Embryonic day 13.5 hindbrain
Embryonic day 14.5 hindbrain
Embryonic day 15.5 forebrain
Embryonic day 15.5 hindbrain
Cortex
FrontalLobe
Cerebellum
Brain (9 organs)
Bladder
Embryonic day 18.5 ovary
Embryonic day 18.5 testis
Postnatal day 28 testis
Postnatal day 63 testis
Testis
Testis (9 organs)
Postnatal day 14 heart
Postnatal day 28 heart
Postnatal day 63 heart
Postnatal day 14 liver
Postnatal day 28 liver
Postnatal day 63 liver
Embryonic day 12.5 liver
Embryonic day 13.5 liver
Embryonic day 16.5 liver
Embryonic day 18.5 liver
Embryonic day 14.5 liver
Embryonic day 15.5 liver
Embryonic day 17.5 liver
Postnatal day 0 liver
Postnatal day 3 liver
Postnatal day 28 kidney
Postnatal day 63 kidney
Liver
Liver (9 organs)
Adrenal
Stomach
Duodenum
SmIntestine
Sample_type
Z-score
4
2
0
−2
−4
Zinc finger divergence time grade
Old
Mid
Young
Sample_type
Early embryo development
Endoderm differentiation
Mesoderm differentiation
Ectoderm differentiation
Early organogenesis to adulthood
Adult tissues or organs

Figure S4E

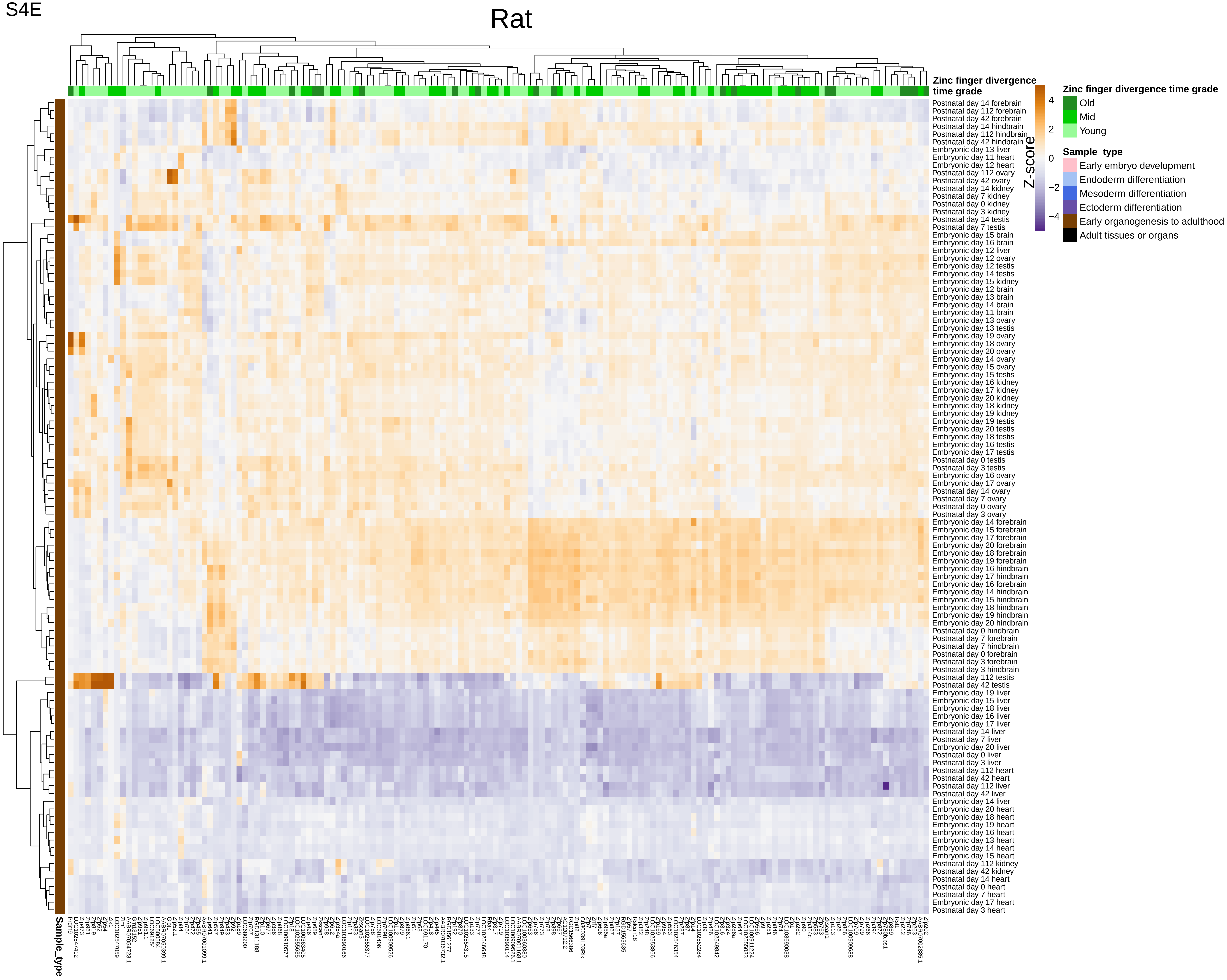
Rat
Zinc finger divergence time grade
Z-score
4
2
0
−2
−4
Zinc finger divergence time grade
Old
Mid
Young
Sample_type
Early embryo development
Endoderm differentiation
Mesoderm differentiation
Ectoderm differentiation
Early organogenesis to adulthood
Adult tissues or organs
Postnatal day 14 forebrain
Postnatal day 112 forebrain
Postnatal day 42 forebrain
Postnatal day 14 hindbrain
Postnatal day 112 hindbrain
Postnatal day 42 hindbrain
Embryonic day 13 liver
Embryonic day 11 heart
Embryonic day 12 heart
Postnatal day 112 ovary
Postnatal day 42 ovary
Postnatal day 14 kidney
Postnatal day 7 kidney
Postnatal day 0 kidney
Postnatal day 3 kidney
Postnatal day 14 testis
Postnatal day 7 testis
Embryonic day 15 brain
Embryonic day 16 brain
Embryonic day 12 liver
Embryonic day 12 ovary
Embryonic day 12 testis
Embryonic day 14 testis
Embryonic day 15 kidney
Embryonic day 12 brain
Embryonic day 13 brain
Embryonic day 14 brain
Embryonic day 11 brain
Embryonic day 13 ovary
Embryonic day 13 testis
Embryonic day 19 ovary
Embryonic day 18 ovary
Embryonic day 20 ovary
Embryonic day 14 ovary
Embryonic day 15 ovary
Embryonic day 15 testis
Embryonic day 16 kidney
Embryonic day 17 kidney
Embryonic day 20 kidney
Embryonic day 18 kidney
Embryonic day 19 kidney
Embryonic day 19 testis
Embryonic day 20 testis
Embryonic day 18 testis
Embryonic day 16 testis
Embryonic day 17 testis
Postnatal day 0 testis
Postnatal day 3 testis
Embryonic day 16 ovary
Embryonic day 17 ovary
Postnatal day 14 ovary
Postnatal day 7 ovary
Postnatal day 0 ovary
Postnatal day 3 ovary
Embryonic day 14 forebrain
Embryonic day 15 forebrain
Embryonic day 17 forebrain
Embryonic day 20 forebrain
Embryonic day 18 forebrain
Embryonic day 19 forebrain
Embryonic day 16 hindbrain
Embryonic day 17 hindbrain
Embryonic day 16 forebrain
Embryonic day 14 hindbrain
Embryonic day 15 hindbrain
Embryonic day 18 hindbrain
Embryonic day 19 hindbrain
Embryonic day 20 hindbrain
Postnatal day 0 hindbrain
Postnatal day 7 forebrain
Postnatal day 7 hindbrain
Postnatal day 0 forebrain
Postnatal day 3 forebrain
Postnatal day 3 hindbrain
Postnatal day 112 testis
Postnatal day 42 testis
Embryonic day 19 liver
Embryonic day 15 liver
Embryonic day 18 liver
Embryonic day 16 liver
Embryonic day 17 liver
Postnatal day 14 liver
Postnatal day 7 liver
Embryonic day 20 liver
Postnatal day 0 liver
Postnatal day 3 liver
Postnatal day 112 heart
Postnatal day 42 heart
Postnatal day 112 liver
Postnatal day 42 liver
Embryonic day 14 liver
Embryonic day 20 heart
Embryonic day 18 heart
Embryonic day 19 heart
Embryonic day 16 heart
Embryonic day 13 heart
Embryonic day 14 heart
Embryonic day 15 heart
Postnatal day 112 kidney
Postnatal day 42 kidney
Postnatal day 14 heart
Postnatal day 0 heart
Postnatal day 7 heart
Embryonic day 17 heart
Postnatal day 3 heart
Sample_type

Figure S4F

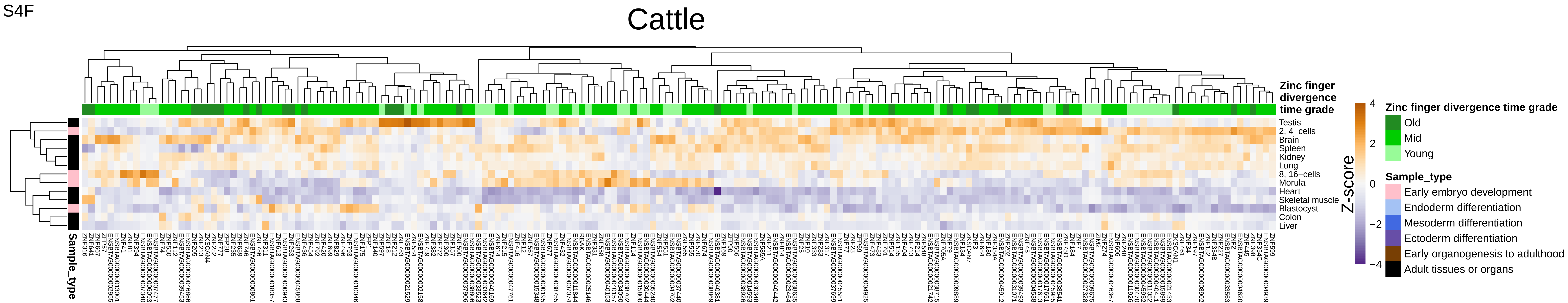
Cattle
Zinc finger divergence time grade
Sample_type
Testis
2, 4−cells
Brain
Spleen
Kidney
Lung
8, 16−cells
Morula
Heart
Skeletal muscle
Blastocyst
Colon
Liver
Z-score
4
2
0
−2
−4
Zinc finger divergence time grade
Old
Mid
Young
Sample_type
Early embryo development
Endoderm differentiation
Mesoderm differentiation
Ectoderm differentiation
Early organogenesis to adulthood
Adult tissues or organs

Figure S4G

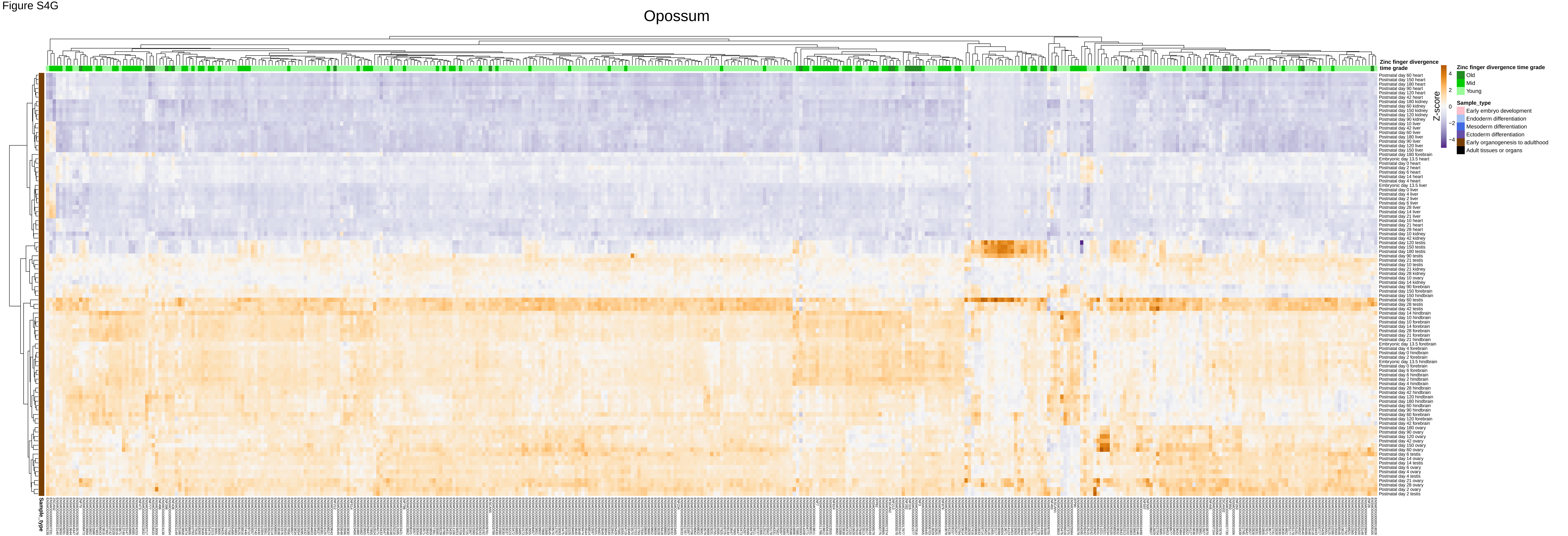
Opossum
Zinc finger divergence time grade
Postnatal day 60 heart
Postnatal day 150 heart
Postnatal day 180 heart
Postnatal day 90 heart
Postnatal day 120 heart
Postnatal day 42 heart
Postnatal day 180 kidney
Postnatal day 60 kidney
Postnatal day 150 kidney
Postnatal day 120 kidney
Postnatal day 90 kidney
Postnatal day 10 liver
Postnatal day 42 liver
Postnatal day 60 liver
Postnatal day 180 liver
Postnatal day 90 liver
Postnatal day 120 liver
Postnatal day 150 liver
Postnatal day 180 forebrain
Embryonic day 13.5 heart
Postnatal day 0 heart
Postnatal day 2 heart
Postnatal day 6 heart
Postnatal day 14 heart
Postnatal day 4 heart
Embryonic day 13.5 liver
Postnatal day 0 liver
Postnatal day 4 liver
Postnatal day 2 liver
Postnatal day 6 liver
Postnatal day 28 liver
Postnatal day 14 liver
Postnatal day 21 liver
Postnatal day 10 heart
Postnatal day 21 heart
Postnatal day 28 heart
Postnatal day 10 kidney
Postnatal day 42 kidney
Postnatal day 120 testis
Postnatal day 150 testis
Postnatal day 180 testis
Postnatal day 90 testis
Postnatal day 21 testis
Postnatal day 10 testis
Postnatal day 21 kidney
Postnatal day 28 kidney
Postnatal day 10 ovary
Postnatal day 14 kidney
Postnatal day 90 forebrain
Postnatal day 150 forebrain
Postnatal day 150 hindbrain
Postnatal day 60 testis
Postnatal day 28 testis
Postnatal day 42 testis
Postnatal day 14 hindbrain
Postnatal day 10 hindbrain
Postnatal day 10 forebrain
Postnatal day 14 forebrain
Postnatal day 28 forebrain
Postnatal day 21 forebrain
Postnatal day 21 hindbrain
Embryonic day 13.5 forebrain
Postnatal day 4 forebrain
Postnatal day 0 hindbrain
Postnatal day 2 forebrain
Embryonic day 13.5 hindbrain
Postnatal day 0 forebrain
Postnatal day 6 forebrain
Postnatal day 6 hindbrain
Postnatal day 2 hindbrain
Postnatal day 4 hindbrain
Postnatal day 28 hindbrain
Postnatal day 42 hindbrain
Postnatal day 120 hindbrain
Postnatal day 180 hindbrain
Postnatal day 60 hindbrain
Postnatal day 90 hindbrain
Postnatal day 60 forebrain
Postnatal day 120 forebrain
Postnatal day 42 forebrain
Postnatal day 180 ovary
Postnatal day 90 ovary
Postnatal day 120 ovary
Postnatal day 42 ovary
Postnatal day 150 ovary
Postnatal day 60 ovary
Postnatal day 6 testis
Postnatal day 14 ovary
Postnatal day 14 testis
Postnatal day 6 ovary
Postnatal day 4 ovary
Postnatal day 4 testis
Postnatal day 21 ovary
Postnatal day 28 ovary
Postnatal day 2 ovary
Postnatal day 2 testis
Sample_type
Z-score
4
2
0
−2
−4
Zinc finger divergence time grade
Old
Mid
Young
Sample_type
Early embryo development
Endoderm differentiation
Mesoderm differentiation
Ectoderm differentiation
Early organogenesis to adulthood
Adult tissues or organs

Figure S5

Sample type	Sample name	Highly expressed KZFPs
Early embryo development	1-cell (1-cell -> Mural trophectoderm)	ZNF560; ZNF727
	2, 4, 8-cell (1-cell -> Mural trophectoderm)	ZNF479; ZNF679; ZNF705D; ZNF705G; ZNF727; ZNF728; ZNF735
	8-cell (2-cell -> ICM)	ZNF479; ZNF679; ZNF705D; ZNF705G; ZNF729; ZNF735
	Morula (1-cell -> Mural trophectoderm)	ZNF729
Reproductive organs (ovary and testis)	11 week post conception ovary	ZNF716
	16 week post conception ovary	PRDM9
	18 week post conception ovary	PRDM9
	18 week post conception testis	ZNF705D
	Young adult testis	ZNF479; ZNF705B
	Middle adult testis	ZNF705B
	Elderly testis	ZNF479
	Testis	PRDM9; ZNF705B; ZNF716; ZNF99
	Testis (GTEx)	ZIM3; ZNF829; PRDM9; ZNF479; ZNF705A; ZNF705G; ZNF716; ZNF730; ZNF99
Brain	12 week post conception forebrain	ZNF860
	Brain - Spinal cord (cervical c-1) (GTEx)	ZFP57
Esophagus	Esophagus	ZNF812
	Esophagus - Mucosa (GTEx)	ZNF812

Figure S6

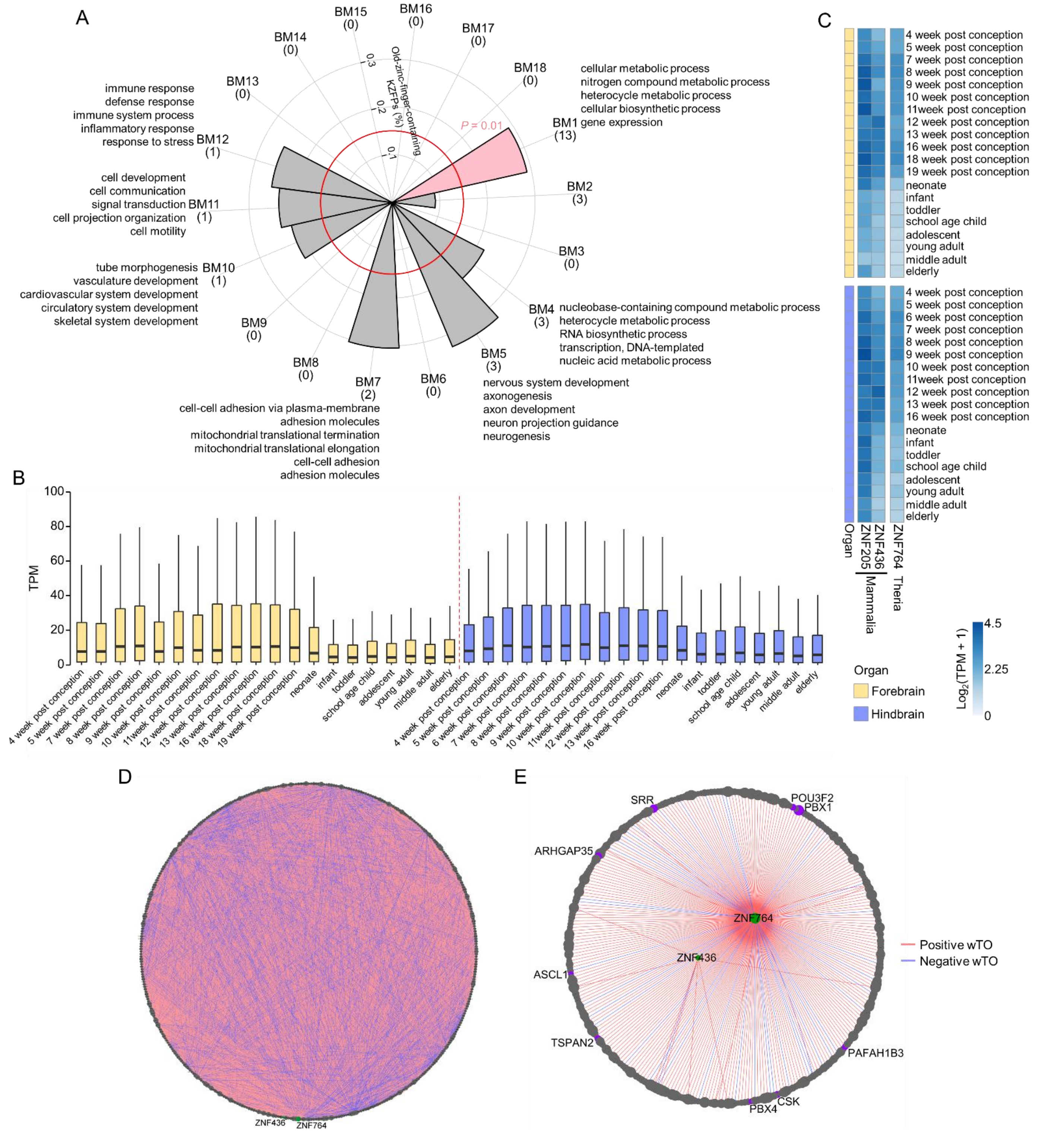
A
BM1 (13)
BM2 (3)
BM3 (0)
BM4 (3)
BM5 (3)
BM6 (0)
BM7 (2)
BM8 (0)
BM9 (0)
BM10 (1)
BM11 (1)
BM12 (1)
BM13 (0)
BM14 (0)
BM15 (0)
BM16 (0)
BM17 (0)
BM18 (0)
Old-zinc-finger-containing KZFPs (%)
0.1
0.2
0.3
P = 0.01
cellular metabolic process
nitrogen compound metabolic process
heterocycle metabolic process
cellular biosynthetic process
gene expression
nucleobase-containing compound metabolic process
heterocycle metabolic process
RNA biosynthetic process
transcription, DNA-templated
nucleic acid metabolic process
nervous system development
axonogenesis
axon development
neuron projection guidance
neurogenesis
cell-cell adhesion via plasma-membrane adhesion molecules
mitochondrial translational termination
mitochondrial translational elongation
cell-cell adhesion
adhesion molecules
tube morphogenesis
vasculature development
cardiovascular system development
circulatory system development
skeletal system development
cell development
cell communication
signal transduction
cell projection organization
cell motility
immune response
defense response
immune system process
inflammatory response
response to stress
B
TPM
0
20
40
60
80
100
4 week post conception
5 week post conception
6 week post conception
7 week post conception
8 week post conception
9 week post conception
10 week post conception
11week post conception
12 week post conception
13 week post conception
16 week post conception
18 week post conception
19 week post conception
neonate
infant
toddler
school age child
adolescent
young adult
middle adult
elderly
C
Organ
ZNF205
ZNF436
ZNF764
Mammalia
Theria
Organ
Forebrain
Hindbrain
Log2(TPM + 1)
4.5
2.25
0
D
ZNF436
ZNF764
E
SRR
POU3F2
PBX1
ARHGAP35
ZNF764
ZNF436
ASCL1
TSPAN2
PAFAH1B3
PBX4
CSK
Positive wTO
Negative wTO

Figure S7

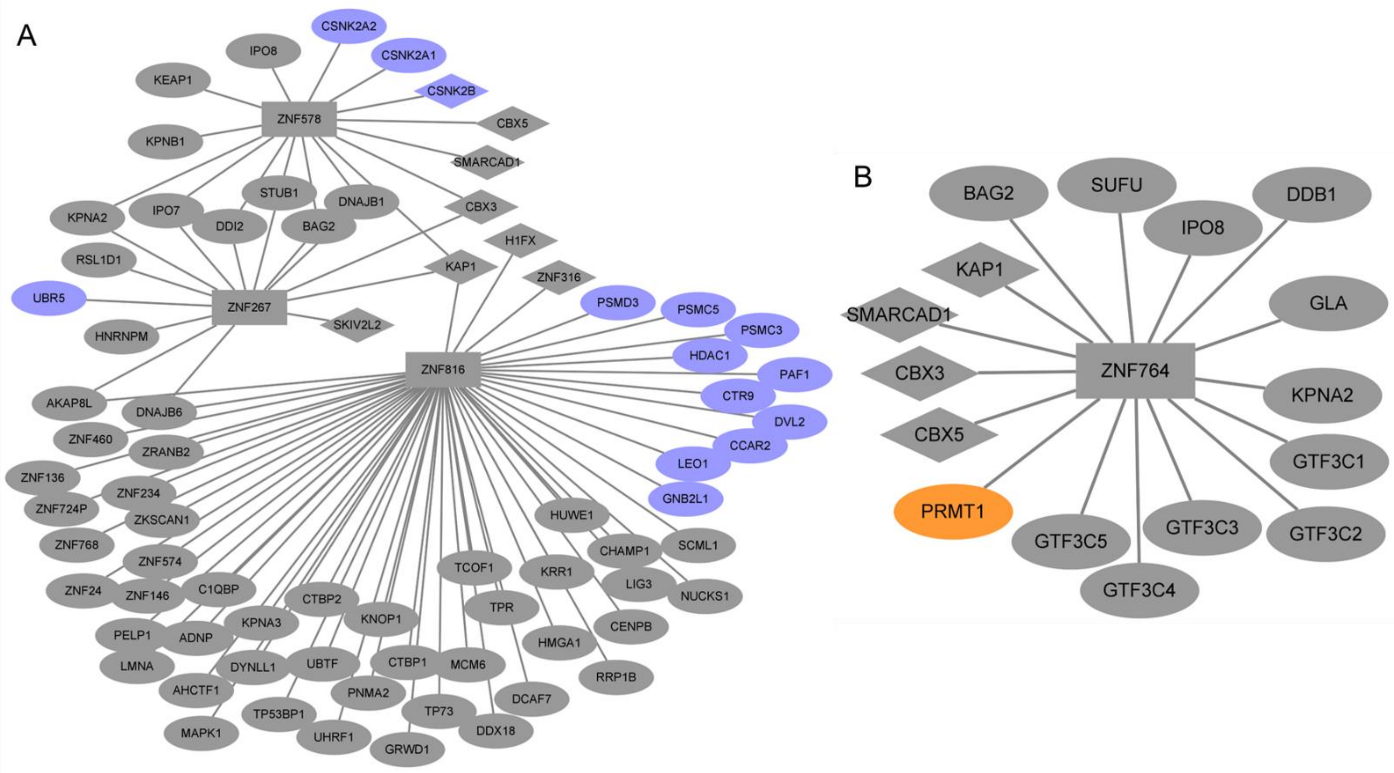
A
ZNF578
ZNF267
ZNF816
CSNK2A2
CSNK2A1
CSNK2B
IPO8
KEAP1
KPNB1
CBX5
SMARCAD1
CBX3
H1FX
KAP1
ZNF316
STUB1
DNAJB1
IPO7
DDI2
BAG2
KPNA2
RSL1D1
UBR5
HNRNPM
SKIV2L2
PSMD3
PSMC5
PSMC3
HDAC1
PAF1
CTR9
DVL2
CCAR2
LEO1
GNB2L1
AKAP8L
DNAJB6
ZNF460
ZRANB2
ZNF136
ZNF234
ZNF724P
ZKSCAN1
ZNF768
ZNF574
ZNF24
ZNF146
C1QBP
CTBP2
KNOP1
KPNA3
ADNP
PELP1
LMNA
DYNLL1
UBTF
CTBP1
MCM6
AHCTF1
PNMA2
TP53BP1
MAPK1
UHRF1
GRWD1
TP73
DDX18
DCAF7
TCOF1
TPR
KRR1
HMGA1
HUWE1
CHAMP1
LIG3
SCML1
NUCKS1
CENPB
RRP1B
B
ZNF764
BAG2
SUFU
IPO8
DDB1
KAP1
SMARCAD1
GLA
CBX3
KPNA2
CBX5
GTF3C1
PRMT1
GTF3C5
GTF3C3
GTF3C2
GTF3C4

Figure S8

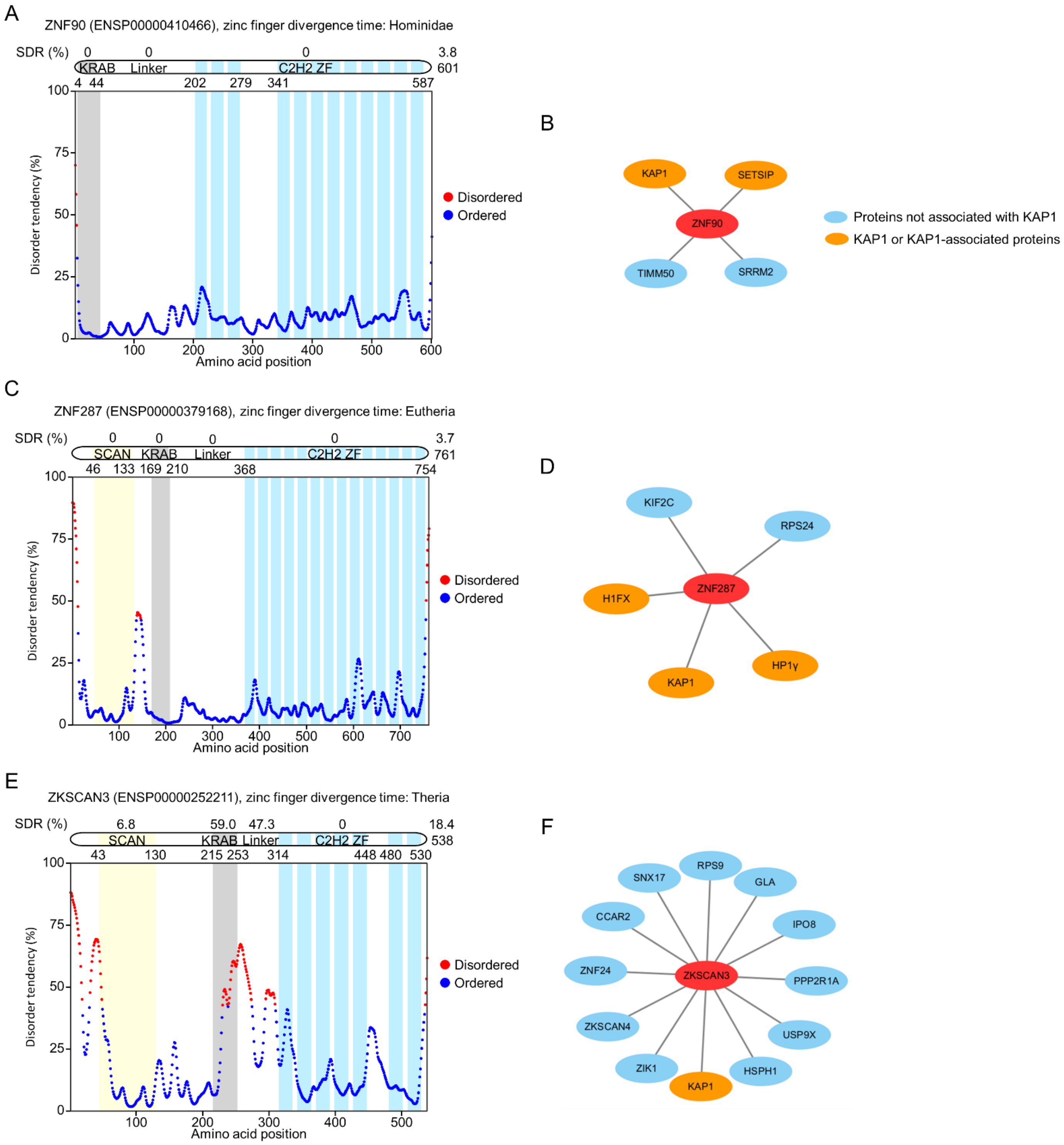

A
ZNF90 (ENSP00000410466), zinc finger divergence time: Hominidae
SDR (%) 0 0 0 3.8
KRAB Linker C2H2 ZF 601
4 44 202 279 341 587
Disorder tendency (%)
Amino acid position
Disordered
Ordered
B
KAP1
SETSIP
ZNF90
TIMM50
SRRM2
Proteins not associated with KAP1
KAP1 or KAP1-associated proteins
C
ZNF287 (ENSP00000379168), zinc finger divergence time: Eutheria
SDR (%) 0 0 0 0 3.7
SCAN KRAB Linker C2H2 ZF 761
46 133 169 210 368 754
Disorder tendency (%)
Amino acid position
Disordered
Ordered
D
KIF2C
RPS24
H1FX
ZNF287
HP1γ
KAP1
E
ZKSCAN3 (ENSP00000252211), zinc finger divergence time: Theria
SDR (%) 6.8 59.0 47.3 0 18.4
SCAN KRAB Linker C2H2 ZF 538
43 130 215 253 314 448 480 530
Disorder tendency (%)
Amino acid position
Disordered
Ordered
F
RPS9
SNX17
GLA
CCAR2
IPO8
ZNF24
ZKSCAN3
PPP2R1A
ZKSCAN4
USP9X
ZIK1
HSPH1
KAP1

Figure S9

A

B

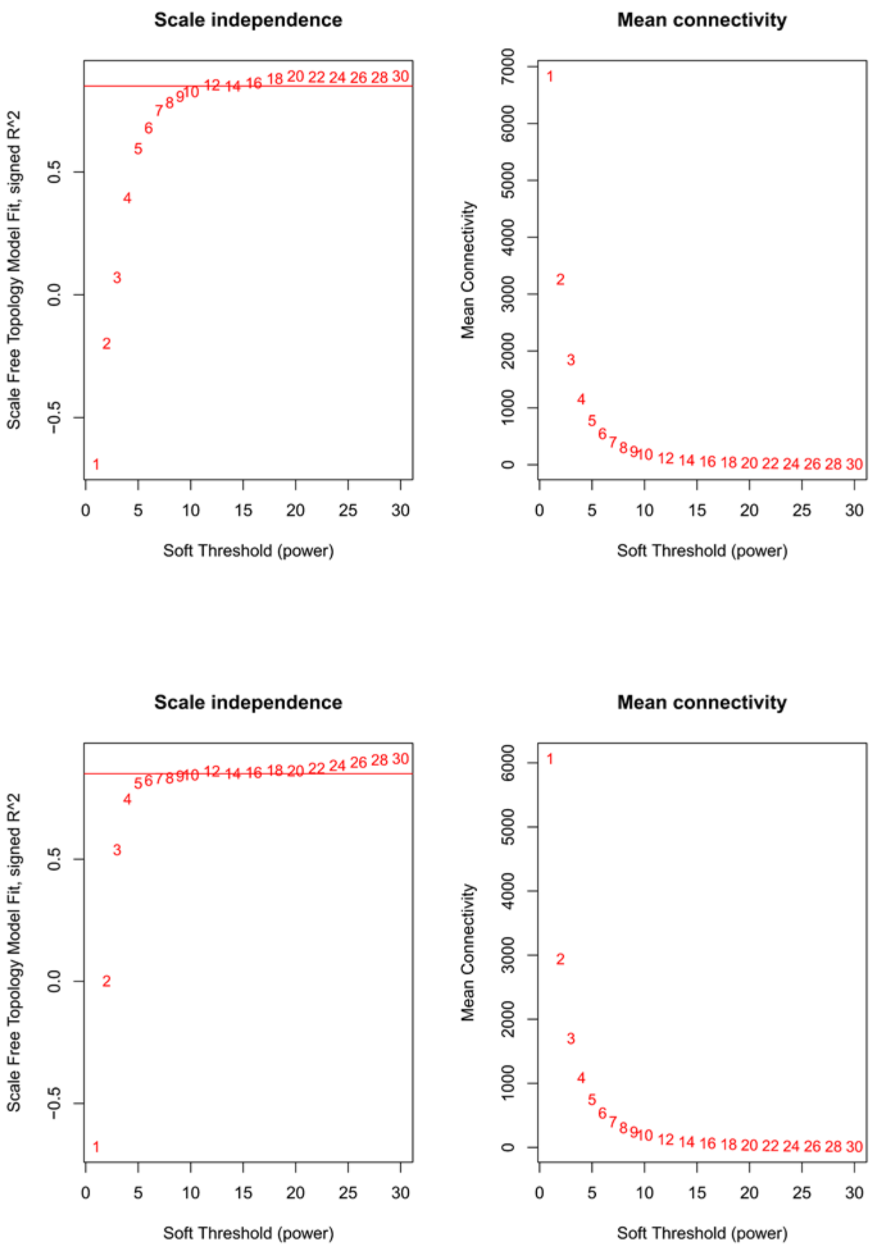

Scale independence
Scale Free Topology Model Fit, signed R^2
Soft Threshold (power)
Mean connectivity
Mean Connectivity
Soft Threshold (power)
Scale independence
Scale Free Topology Model Fit, signed R^2
Soft Threshold (power)
Mean connectivity
Mean Connectivity
Soft Threshold (power)
